# Model-based cardiac field artefact correction for OP-MEG

**DOI:** 10.1162/IMAG.a.1353

**Published:** 2026-09-03

**Authors:** Sascha P. Woelk, Nicholas A. Alexander, George C. O’Neill, Sarah N. Garfinkel, Gareth R. Barnes

**Affiliations:** Institute of Cognitive Neuroscience, University College London, London, United Kingdom; Department of Imaging Neuroscience, Institute of Neurology, University College London, London, United Kingdom; Department of Neuroscience, Physiology and Pharmacology, University College London, London, United Kingdom

**Keywords:** OP-MEG, magnetoencephalography, artefact removal, cardiac field artefact, cardiac cycle effects, interoception

## Abstract

Each heartbeat creates an electro-magnetic field that propagates throughout the body and is superimposed on measured brain activity in electroencephalography (EEG) and magnetoencephalography (MEG) recordings. Being several orders of magnitude larger than a typical evoked response in the brain, the Cardiac Field Artefact (CFA) is especially problematic in studies where cortical activity time locked to the cardiac cycle is of interest, such as in interoception research. In this work, we develop a model-based CFA correction method, which exploits the fact that the magnetic field propagates relatively evenly through the mostly diamagnetic tissue of the human body, and that in studies using wearable optically pumped magnetometers (OP-MEG), participants are free to rotate their head. Free head rotation decouples the heart’s magnetic field from that of the brain, allowing for the creation of a cardiac dipole moment (CDM) estimate, that is, an estimate of the electrical current flow in the heart, which is minimally confounded by concurrent neuronal activity in the brain. We show in simulations that, given information about the relative position and orientation of head and chest, this CDM estimate can be used to predict and correct the sensor-level CFA by dynamically accounting for the spatial relationship between heart, brain, and sensors. We further provide an empirical proof-of-principle demonstration of the pipeline in a single participant using recordings with intermittent head movement, tracked by optical motion capture, as well as an established auditory response paradigm with well-characterized response pattern.

## 1 Introduction

### 1.1 The cardiac field artefact

With each beat, the heart generates a powerful electro-magnetic field that propagates along the body, including to the scalp. Its appearance in electroencephalography (EEG) and magnetoencephalography (MEG) recordings has been termed the cardiac field artefact (CFA) (Dirlich et al., 1997).

The CFA is most prominent during the QRS complex and T wave of the cardiac cycle, and its appearance in the recorded field patterns on the scalp is distinctly asymmetrical, especially at the time of the R wave and T wave (Dirlich et al., 1997). It has been shown that the CFA affects a large number of sensors, and is highly correlated with the electrocardiogram (ECG) time series, particularly in frequencies up to 30 Hz (Brookes et al., 2011).

The CFA is especially problematic when cortical activity time locked to the cardiac cycle is being measured—for example, in the growing research field of interoception. Interoception research investigates how afferent signals from within the body, including from the heart, are integrated by the nervous system at various conscious and subconscious levels (Azzalini et al., 2019; Critchley & Harrison, 2013; Suksasilp & Garfinkel, 2022). A common research paradigm in cardiac interoception is to present stimuli either at systole (i.e., the time when the heart contracts and baroreceptors inform the brain about cardiac activity) or at diastole (i.e., the time when the heart relaxes and baroreceptors are quiescent) and to study their differential processing and behavioural responses (Al et al., 2020; Arslanova et al., 2023; Critchley & Harrison, 2013; Garfinkel & Critchley, 2016; Garfinkel et al., 2013).

Another area of interoception research, where the CFA obscures potentially valuable measurements, is the study of heartbeat-evoked responses, often termed heartbeat-evoked potentials (HEP; Schandry et al., 1986). HEPs are neural responses time locked to the cardiac cycle and are thought to reflect the cortical processing of cardiac signals (Park & Blanke, 2019). Abnormalities in the HEP have been linked to various mental health conditions, functional seizures, chronic pain, and stress (Elkommos et al., 2023; Park & Blanke, 2019; Schulz & Vögele, 2015; Solcà et al., 2020). However, basic properties underlying the HEP remain unexplored and there is no common standard of measurement (Coll et al., 2021).

### 1.2 Existing methods for CFA correction

One of the simplest approaches to artefact correction is event-related averaging of data to an experimental stimulus, which can ameliorate the impact of many artefacts, including the CFA (Luck, 2014; Sun et al., 2016). However, this approach is not suitable when the activity under study itself is time locked to the cardiac cycle, because averaging to the R-peak of the cardiac cycle would reinforce, instead of ameliorate the CFA (Dirlich et al., 1997; Stephen, 2019).

Equally, subtraction and regression approaches are challenging for CFA correction and have received limited research attention in this context. This is because the direction and magnitude of cardiac current flow vary dynamically during the cardiac cycle, which renders the problem non-linear (Dirlich et al., 1997; Gulrajani et al., 1984). Moreover, in the context of EEG, the rules of propagation of electrical current from the thorax to the scalp are highly complex and difficult to model mathematically (Dirlich et al., 1997). In the future, machine learning techniques may help to advance regression-based approaches to CFA correction, as evidenced by a recent study that employed neural network regression to successfully correct the CFA in EEG data (Arnau et al., 2023). However, a key challenge with machine learning techniques is their limited interpretability: the underlying mechanisms are often opaque (e.g., distributed across the layers of a deep neural network) (Antamis et al., 2024), making it challenging to evaluate their validity in the context of a phenomenon such as the HEP, for which no uniform definition and standard of measurement has yet been established (Coll et al., 2021; Virjee et al., 2025).

Because of these challenges, common methods for CFA correction include mainly signal separation techniques such as independent component analysis (ICA), principal component analysis (PCA) (Taulu et al., 2004), signal-space projection (SSP; Uusitalo & Ilmoniemi, 1997), spectral SSP (Ramírez et al., 2011), as well as signal-space separation (SSS; Taulu et al., 2004); while most recent progress in CFA correction has been in the area of automated ICA procedures (Breuer et al., 2014; Campos Viola et al., 2009; Issa et al., 2019; Mantini et al., 2008; Pion-Tonachini et al., 2019; Radüntz et al., 2017; Tamburro et al., 2019). Although generally effective, these methods rely primarily on statistical and spatial separation rather than on an explicit model of the underlying cardiac source activity, and may, therefore, not completely remove the CFA from the recorded data, while running the risk of removing parts of the brain signal of interest (Stephen, 2019).

### 1.3 The present study

In the present study, we present a novel, model-based method for CFA correction, which exploits the unique advantages of optically pumped magnetoencephalography (OP-MEG). OP-MEG refers to a new generation of wearable MEG systems, which are lightweight and allow participants to move relatively freely during recording sessions (Boto et al., 2018; Seymour et al., 2021; Tierney et al., 2019).

The key point is that by moving the head during OP-MEG recordings, the data recorded from the brain become decoupled in space from the magnetic fields generated by the heart (as illustrated in Fig. 2). While traditional cryogenic MEG theoretically allows for similar experimental designs by instructing participants to rotate the torso while keeping the head stationary relative to the sensor array (e.g., see Wang et al., 2016), the unique advantage of OP-MEG systems is that participants can move both head and body throughout recording sessions, enabling more comfortable and naturalistic experiments.

By making use of the variable geometrical relationship between brain and heart, it is possible to derive an estimate of the current flow in the heart which is minimally confounded by concurrent neuronal activity. Since cardiac activity can be well described in a three-dimensional vector space (Dirlich et al., 1997; Gulrajani et al., 1984), it becomes mathematically tractable to predict how the magnetic field generated by the heart projects onto the OP-MEG sensor array on the scalp, for any head rotation. This estimate of cardiac current flow can then be used to predict the heart-generated magnetic field, dynamically accounting for the spatial relationship between thorax and scalp. This is feasible because magnetic fields are only mildly attenuated by the mostly diamagnetic tissue of the human body (Hamalainen & Sarvas, 1987, 1989; Okada et al., 1999), allowing the tissues of the upper body (heart, lungs, and torso) to be approximated with a relatively simple, piecewise homogeneous volume conductor model (Mäntynen et al., 2014). In contrast, applying the same idea to EEG studies is more challenging, because unlike the magnetic field, the measured electric potential is shaped by complex tissue conductivity properties, illustrated by the fact that the electrical potential difference due to the heart at the scalp changes to only a maximum of 45% of the angle by which the head is rotated (Dirlich et al., 1997).

We show, using simulations and a proof-of-principle empirical dataset, that it is possible to create this estimate of cardiac current flow with a small number of current dipoles at the location of the heart. In the following section, we discuss the theoretical model underlying the proposed CFA-correction method. We then introduce our methodological approach for validation of the method via simulations, as well as the experimental setup for empirical validation, followed by presentation of the results.

## 2 Theory

### 2.1 Modelling the heart’s far field with equivalent current dipoles

The magnetic field measured by an OP-MEG sensor array reflects the superposition of multiple concurrently active sources. In the simplified model considered here, the total measured field, *B_measured_* ∈ ℝ*^m×t^*, arising from concurrent cardiac and brain activity, recorded with *m* channels at *t* time points, can be expressed as follows:

$$B_{measured}=B_{Heart}+B_{Brain}$$ (1)

To model the cardiac contributions to the measured field, assumptions about the underlying cardiac source configuration are required. In medical magnetocardiography (MCG), a distributed source model is usually applied to be able to describe the full complexity of the heart’s activity (Mäntynen et al., 2014). However, the far field (such as that recorded at the scalp by an array of OP-MEG sensors) can be approximated with only a small number of equivalent current dipoles (ECD; Scherg, 1990), because the large distance between the heart and the scalp sensors causes spatially distributed cardiac sources to sum up to a much simpler (dipolar) pattern at the sensor level. Allowing the orientations of these dipoles to vary freely enables the model to account for changes in direction and magnitude of cardiac current flow throughout the cardiac cycle (Gulrajani et al., 1984).

Given a volume conductor model of the torso, information about the sensor geometry, and an estimate of the heart’s location, a lead field matrix can be computed that defines how current flow at the heart’s location translates into measured magnetic fields (Baillet, 2010).

The forward model for the heart’s electromagnetic activity as measured by an array of magnetometers or OP-MEG sensors on the scalp can thus be expressed as

$$B_{Heart}=L_{Heart}S_{Heart}+\varepsilon ,$$ (2)

where *S_Heart_* ∈ ℝ*^v×t^* contains the time-varying source activations associated with the depolarization and repolarization of the cardiac muscle across *t* time points. Each cardiac source is represented by three orthogonal dipole-orientation components (*x*, *y*, *z*), such that *v* = 3*d*, with *v* denoting the total number of dipole-orientation components and *d* denoting the number of cardiac sources modelled. *L_Heart_* ∈ ℝ*^m×v^* represents the lead field matrix which provides the spatial mapping from the *v* dipole-orientation components to *m* OP-MEG channels. Note that typically we use triaxial OP-MEG sensors, each with three sensitive axes; here we refer to each axis as a channel. Finally, ε models independent channel-level noise.

The brain’s electromagnetic activity can be expressed analogously as

$$B_{Brain}=L_{Brain}S_{Brain}+\varepsilon ,$$ (3)

where *S_Brain_*, *L_Brain_*, *B_Brain_*, and ε are defined in the same way as for cardiac sources.

### 2.2 Correction of the CFA

Given the additive nature of the measured magnetic fields, it should theoretically be possible to obtain an artefact-free brain signal by subtracting the predicted cardiac field from the measured field. The challenge is thus to accurately model the CFA.

The unique advantage of OP-MEG is that participants can freely rotate their head during recording sessions. When the coordinate system is centred with regard to the position of the torso, the measured field from the heart *B_Heart_* stays fixed in space independent of the head rotation angle, while the measured field from the brain’s neural activity *B_Brain_* rotates with the head. Estimating cardiac source activity across multiple head rotations, therefore, improves the estimate of the CFA while reducing contamination from concurrent brain activity.

Our CFA-correction method estimates cardiac current flow using training data (i.e., on-scalp OP-MEG measurements) from multiple head rotations. A participant-specific estimate of the cardiac dipole moment (CDM) is constructed as follows:

$$CDM_{estimate}=L_{\;all}^{+}B_{all},$$ (4)

where *B_all_* ∈ ℝ*^(m×r)×t^* contains the training data concatenated across *r* head rotations, measured by *m* channels at *t* time points per rotation. $L_{all}$ ∈ ℝ*^(m×r)×v^* is the corresponding concatenated lead field matrix, and $L_{\;all}^{+}$ ∈ ℝ*^v×(m×r)^* its Moore–Penrose pseudoinverse. The product of $L_{\;all}^{+}\;$ and *B_all_* yields the $CDM_{estimate}$ ∈ ℝ*^v×t^*, the estimate of the dipole moment of *d* cardiac sources at all time points *t* in the QRS complex. For a single cardiac source and three head rotations, *B_all_* has 3* m* rows and *t* columns, $L_{\;all}^{+}\;$ has three rows and 3* m* columns, and the resulting $CDM_{estimate}$ has three rows and *t* columns.

Mathematically, Equation (4) computes the least-squares solution to the stacked forward model *B_all_ = L_all_ CDM* + *ε*, that is, it finds a single CDM estimate shared across head rotations that minimizes the mismatch between predicted and measured fields (i.e., *CDM_estimate_* = argmin*_CDM_ ||L_all_ CDM – B_all_||^2^*). Concatenating *r* head rotations increases the number of stacked sensor equations (rows) from *m* to *m × r*, while the number of unknown dipole-orientation components stays fixed (e.g., *v* = 3 for a single cardiac source with three dipole orientation components). This constrains the solution and improves the stability of the CDM estimate. By contrast, brain activity produces rotation-dependent sensor topographies that are inconsistent with a single CDM across rotations and, therefore, gets captured in the residual *ε* = *B_all_ – L_all_ CDM_estimate_.*

The resulting CDM estimate can be used to correct unseen experimental data as follows:

$$\hat{CFA}=L_{unseen}CDM_{estimate,}$$ (5)

$$B_{corrected}=B_{uncorrected}-\hat{CFA},$$ (6)

where *L_unseen_* ∈ ℝ*^m×v^* denotes the lead field matrix for the unseen head rotation, $\hat{CFA}$ ∈ ℝ*^m×t^* denotes the predicted CFA as measured by the OP-MEG channels, *B_uncorrected_* ∈ ℝ*^m×t^* denotes the uncorrected training data, and *B_corrected_* ∈ ℝ*^m×t^* denotes the corrected data. That is, the CDM estimate is projected through the lead field matrix of the unseen head rotation to predict the CFA at sensor level. This prediction is then subtracted from the data, yielding the CFA-corrected data.

## 3 Methods

### 3.1 Simulations: Methods

As the first step, we validated the theoretical model via simulations.

#### 3.1.1 OP-MEG sensor simulation

We used SPM’s *spm_opm_sim* function (Litvak et al., 2011) to simulate an OP-MEG sensor array on the SPM template brain (*single_subj_T1.nii*) (for a detailed description of the approach see Mellor et al., 2024). The simulated array had 85 single-axis OP-MEG sensors with sensor spacing of 35 mm and scalp-to-sensor distance of 15 mm, reflecting a typical OP-MEG sensor array configuration used in studies in our laboratory.

#### 3.1.2 Volume conduction model of the torso

**Fig. 1.**
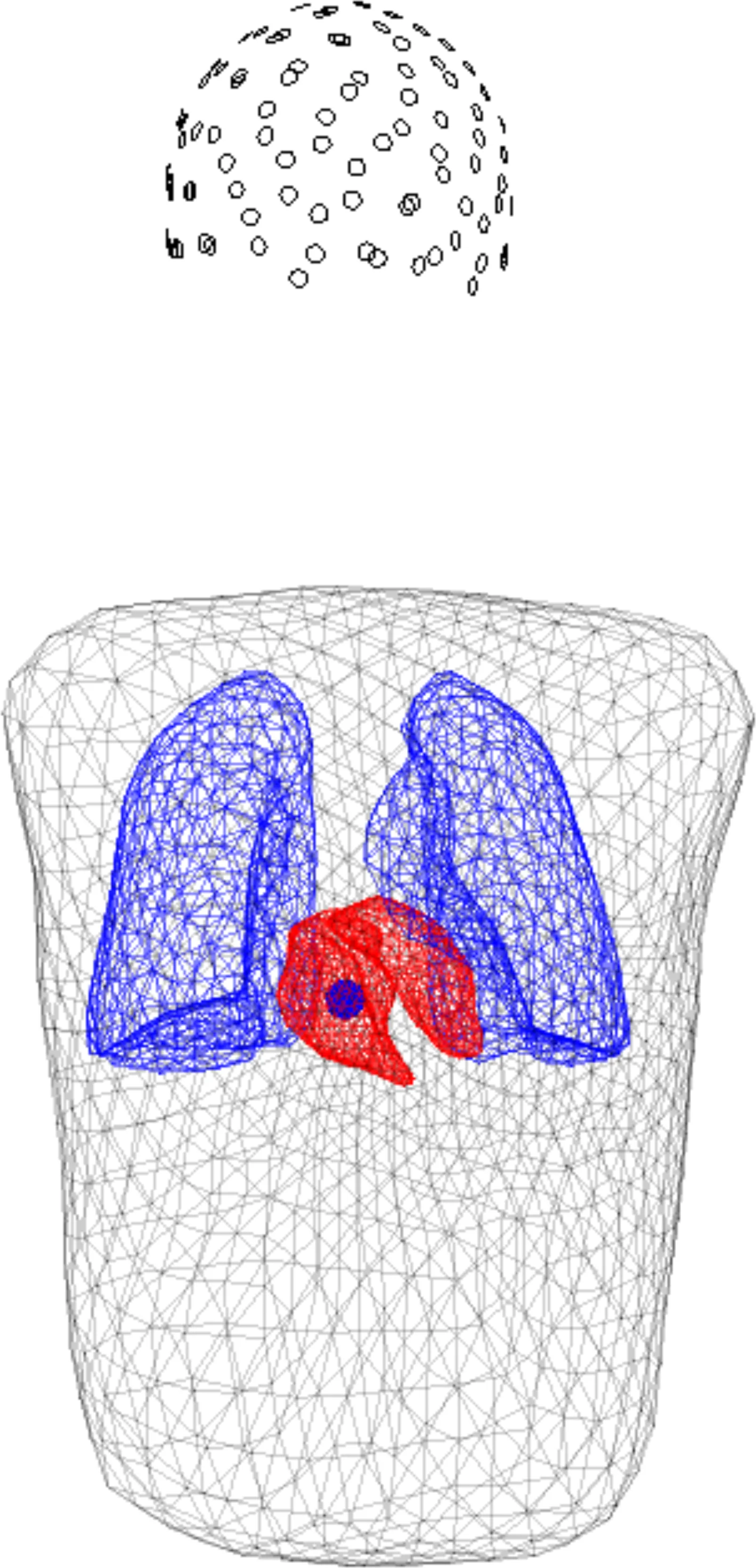
Three-compartment volume conductor model of the torso, including the major conductive volumes of the heart, lungs, and torso. The simulated OP-MEG sensor array is displayed above the torso. The location of the equivalent current dipole (ECD) used to model the cardiac source current is indicated by the point inside the heart compartment.

Leveraging prior work showing that magnetocardiographic fields are influenced by nearby tissue conductivity inhomogeneities (Bruder et al., 1994; Mäntynen et al., 2014), we employed a realistically shaped, piecewise-homogeneous, three-compartment (heart, lungs, torso) volume conductor model (Fig. 1) from the torso tools toolbox (https://github.com/FIL-OPMEG/torso_tools; O’Neill et al., 2025). The model was restricted to the thorax, with more distant anatomy such as the head and arms omitted as a simplifying approximation. Supplementary simulations comparing models with and without the head showed that its inclusion had no effect on the correction performance (*t*(74) = −1.76, *p* = .082). For details, refer to Supplementary Material 1.

The compartments of the heart (i.e., intra-cardiac blood), lungs, and torso were assumed to be homogeneous with conductivities of 0.62, 0.05, and 0.23 S/m (Gabriel et al., 2009; Mäntynen et al., 2014). The volume conduction model was computed using the boundary element method (BEM) following the Helsinki BEM framework (Stenroos & Nummenmaa, 2016; Stenroos et al., 2014) as implemented in the torso tools toolbox.

#### 3.1.3 Simulation of the heart’s electromagnetic activity

The heart’s electromagnetic activity was modelled as an ECD inside the myocardium. The location of this dipole was fixed at the centre of the largest compartments of the triangulated surface model of the heart. For this, we selected the centre point of the first compartment extracted from the heart mesh with the *spm_mesh_clusters* function (Litvak et al., 2011). The compartment centre point was defined as the mean of all vertex positions of this compartment. The unit dipole was oriented in the inferior direction, pointing from the head toward the hips (i.e., approximately following the major axis of depolarization of the cardiac muscle). Then, the lead field matrix *L_Heart_* ∈ ℝ*^m×v^* for the heart dipole, which defines the spatial mapping from the cardiac source space to the OP-MEG sensor space, was computed using the FieldTrip function *ft_prepare_leadfield* (Oostenveld et al., 2011).

To ascertain the expected signal topographies of the heart’s electromagnetic activity at the level of the OP-MEG sensor array on the scalp, we projected a one-dimensional cardiac dipole moment *S_Heart_* ∈ ℝ*^1×t^* of length *t* via the lead field matrix *L_Heart_* to obtain the simulated OP-MEG channel readings. As the test signal, we used a single 1-second QRS complex waveform, sampled at 250 Hz, extracted from a standard ECG recording and adjusted the dipole amplitude so that the measured field had a peak amplitude of approximately 3 pT, matching the CFA amplitude observed in the empirical data from our test recording. The simulated data *B_Heart_* ∈ ℝ*^m×t^* of the heart’s magnetic field contained measurements from *m* = 85 channels across *t* = 250 time points.

In the next step, we added a reference signal to the simulated cardiac signal to serve as a benchmark for assessing CFA correction performance. Specifically, a reference signal matrix *B_Reference_* ∈ ℝ^m×t^ with the same dimensions as the simulated data (i.e., m = 85 channels × t = 250 time points) was generated using normally distributed pseudorandom values with zero mean and unit standard deviation. The signal was scaled to 10% of the absolute peak amplitude of the simulated cardiac signal *B_Heart_*, consistent with previous research showing that cardiac magnetic fields typically exceed cortical signals by approximately one order of magnitude (Hämäläinen et al., 1993). Within each channel, the reference time series was kept identical across trials. In contrast to simulating a specific cortical source, this approach did not impose a physiologically dependent spatial covariance structure across sensors and thus avoided source-specific interactions with the tested CFA correction methods. It, therefore, allowed CFA correction performance to be evaluated independently of a specific cortical topography.

#### 3.1.4 Construction of the CDM estimate from simulation data

To construct the CDM estimate, we simulated training data for multiple head rotations. The simulated training data from all head rotations *r* were then concatenated into a single training data matrix *B_all_* ∈ ℝ*^(m×r)×t^* where *m* = 85 channels and *t* = 250 time points. We varied *r* to test whether using data from a greater number of head rotations could improve the accuracy of the CDM estimate. For each head rotation, a lead field matrix was calculated to project the field-generated CDM (i.e., the cardiac source current) onto the sensor space of the OP-MEG scalp array. The lead field matrices from all head rotations *r* were concatenated in the same order as the training data, resulting in a single lead field matrix *L_all_* ∈ ℝ*^(m×r)×v^* where *m* = 85 channels and *v* is the number of dipole-orientation components (i.e., *v* = 3 for the model with a single dipole *d*). Once the training data and all corresponding lead fields were defined, the CDM estimate was computed according to Equation (4).

#### 3.1.5 Testing the accuracy of the CDM estimate

We assessed the accuracy of the CDM estimate by using it to correct a simulated test dataset, generated from an unseen head rotation. The test rotation was 0˚ (facing forward), which was excluded from the training set.

To assess the benefit of including multiple distinct head rotations in CDM estimation, we performed simulations using CDM estimates derived from training sets containing from *r* = 1 to *r* = 50 distinct head rotations. In each simulation run, *r* head rotations were randomly sampled without replacement from a candidate set ranging from 100˚ leftward to 100˚ rightward, in 4˚ increments, excluding 0˚. Following the methodology described above, simulated training data were generated for the sampled rotations, and the concatenated training data and lead field matrices were used to estimate the dipole moment of the heart, following Equation (4), yielding one CDM estimate per simulation run.

To assess accuracy, we examined how well each CDM estimate recovered the test signal. For each simulation run, the CDM estimate was projected to sensor level via the lead field matrix of the unseen test rotation, yielding the CFA prediction according to Equation (5). This prediction was then used to correct the test signal following Equation (6). Accuracy was quantified as the root mean square error (RMSE) between the corrected data and the reference signal. For each training set size, this procedure was repeated 100 times with a different random sample of rotation angles on each iteration, and the resulting RMSE values were averaged to obtain one mean RMSE per training set size.

#### 3.1.6 Recovering brain activity that is time locked to the cardiac cycle

After testing the general performance of the CFA-correction method, we proceeded to test whether we could successfully recover a simulated brain signal time locked to the cardiac cycle.

For this purpose, we simulated an event-related field (ERF) at the location of the right insula (MNI152 coordinates: *x* = 35 mm, *y* = -11 mm, *z* = 6 mm; Deen et al., 2011). We selected the insula, because it is a key brain region for sensing, interpreting, and integrating information about the inner state of the body, including cardio-afferent signals (Craig, 2009; Critchley et al., 2004; Hsueh et al., 2023; Khalsa et al., 2009).

The volume conduction model for the head was computed based on the inner cortical surface mesh (*cortex_5124*) from the SPM template brain (Litvak et al., 2011), using the corrected-sphere single-shell method (Nolte, 2003) as implemented in FieldTrip (Oostenveld et al., 2011). Using this volume conduction model, the lead field matrix *L_Brain_* ∈ ℝ*^m×3^* was computed using FieldTrip’s *ft_prepare_leadfield* function, containing the spatial mapping between the three orthogonal source orientation components (*x*, *y*, *z*) of the insular brain source to the *m* = 85 channels of the OP-MEG scalp sensor array.

To generate the insula ERF, we used a Gaussian waveform of equal length as the cardiac source signal (i.e., 1 second sampled at 250 Hz), centred at the peak of the R-wave of the QRS complex (i.e., exactly coinciding with the heartbeat). The insula source signal *S_Brain_* ∈ ℝ*^1×t^* had length *t = 250 time points* and its peak dipole moment was set to 0.03% of the maximum absolute amplitude of the cardiac source dipole moment *S_Heart_*, reflecting that typical evoked fields in the brain measure only several tens to hundreds of femtotesla (fT), while the amplitude of the QRS complex is several tens of picotesla (pT) (Hämäläinen et al., 1993). The source signal was projected to sensor level using the weighted sum of the three orthogonal lead field components, with equal weights assigned to each component. The resulting simulated measurement data *B_Brain_* ∈ ℝ*^m×t^* of the right insula ERF contained data from *m* = 85 channels at *t* = 250 time points.

To obtain the final test dataset, we added the simulated insula activity to the previously generated test data containing only cardiac signal and noise.

We then corrected these test data using a CDM estimate built with training data from 20 head rotations. To evaluate CFA-correction performance, we computed the RMSE between the corrected brain signal and the simulated insula activity. For better interpretability, we normalized the RMSE by the amplitude range of the simulated insula ERF.

#### 3.1.7 Robustness of the CDM estimate to cardiac source misplacement in the forward model

Unless a structural MRI scan of the participant’s torso is available, the exact location of the participant’s heart will be unknown. This means there is a risk of misplacement of the ECD representing the heart. Therefore, we simulated the impact of misplacement of the cardiac source dipole in the forward model. We also tested whether modelling the heart with multiple dipoles can increase the robustness of the CDM estimate to such errors, given that it has previously been shown that the signal topographies of an ECG/MCG source distribution can be approximated with a linear combination of ECDs (Mäntynen et al., 2014).

Specifically, we tested the relative performance of a CDM estimate built with two dipoles, compared with an estimate with a single dipole. Six linearly spaced distances between 0 and 50 mm from the “true” heart location (i.e., the centre point of the heart mesh in the volume conductor model) were tested.

For each of these distances, 10 simulation runs were performed. During each run, a randomly oriented unit vector, originating from the centre point of the heart mesh, was computed and scaled by the distance value. The end point of the vector determined the dipole location for the current run. The heart compartment in the volume conductor model was moved by the same distance. This was intended to simulate that without knowledge of the true heart location, a researcher might model both heart source and heart compartment away from the true heart location. Using the new dipole location, a CDM estimate was constructed and tested by correcting the simulated test data. Correction accuracy was quantified by computing the RMSE between the corrected data and the reference signal. RMSE values were averaged across runs to derive an average accuracy metric per distance value.

For the two-dipole model, the same process was employed, with the addition of a second dipole location. Similar to the first dipole, the location of the second dipole was set using a second randomly oriented unit vector, scaled by the same distance as the first unit vector.

#### 3.1.8 Evaluation of the validity of a standardized torso volume conductor model

To examine the effect of employing a standardized volume conductor model for the torso, instead of a personalized torso model, we performed simulations in which the original torso volume conductor model was morphed by independently scaling height and transverse body dimensions by ±20%, yielding a total of eight additional torso anatomies. The heart and lung meshes were transformed jointly with the torso to preserve internal anatomical consistency.

With each of these morphed torso models, 100 simulation runs were performed, each with a different set of head rotations utilized for CDM estimation. Specifically, in each simulation run, a CDM estimate was constructed by utilizing the data from 10 head rotations randomly sampled without replacement from a candidate set ranging from 100˚ leftward to 100˚ rightward, in 4˚ increments, excluding 0˚. The resulting CDM estimates were used to correct the CFA in simulated data generated with the original torso model (i.e., the ground truth), using either a one- or two-dipole solution. The error due to torso geometry was quantified by computing the RMSE between the corrected signal and the reference signal at time of the R-peak, and averaged across runs.

### 3.2 Empirical measurement: Methods

After establishing the feasibility of the CFA-correction approach in simulations, we evaluated the pipeline in an empirical proof-of-principle experiment.

#### 3.2.1 Participant information

The study was approved by the University College London Research Ethics Committee (approval number 3090 0005) and conducted in line with the Declaration of Helsinki. One healthy male participant aged 57 years participated in the study. The participant provided written informed consent.

#### 3.2.2 OP-MEG data acquisition

**Fig. 2.**
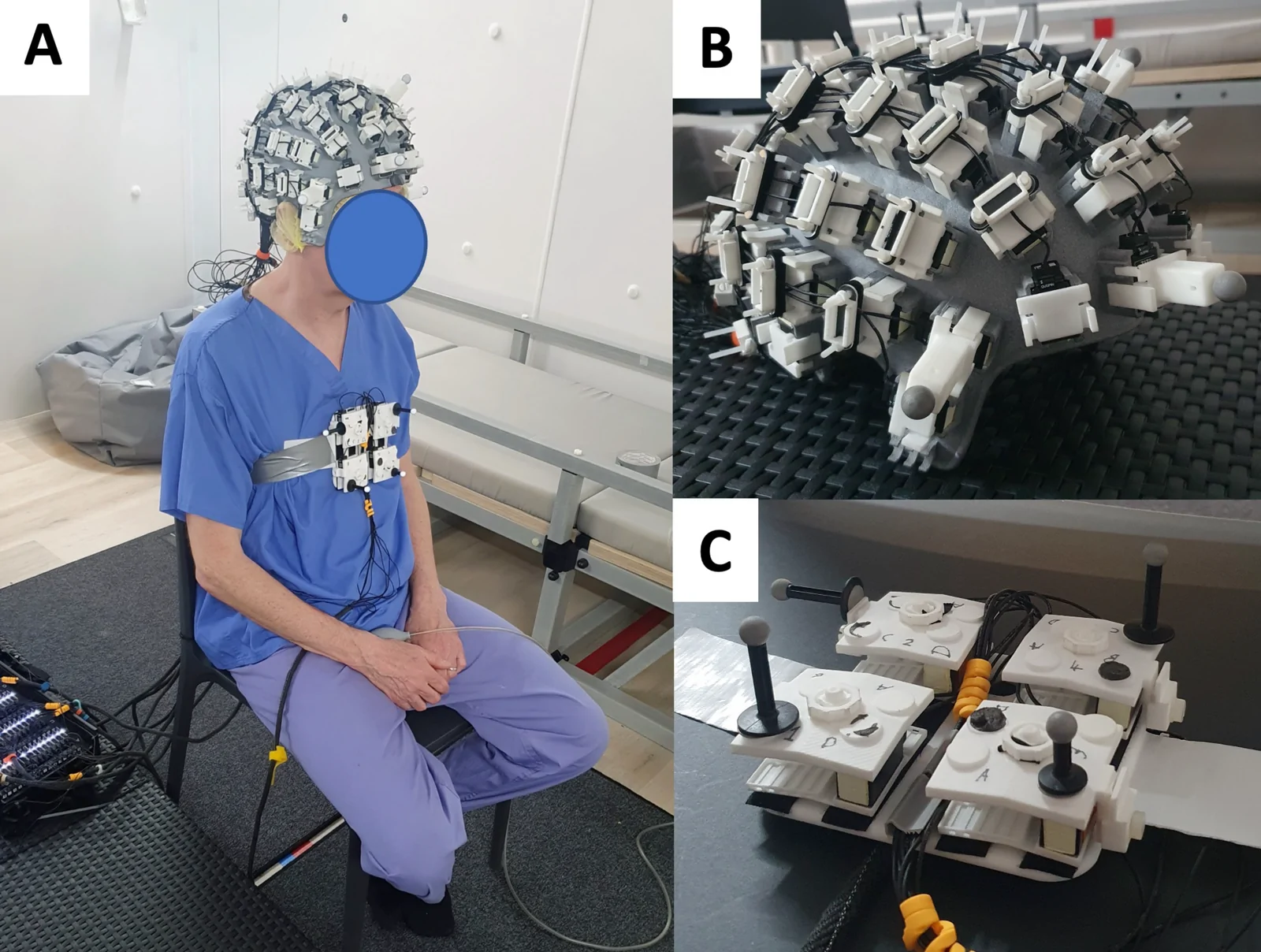
The experimental setup. (A) The participant is seated in the centre of the magnetically shielded room, wearing OP-MEG sensor arrays on scalp and chest. (B) The scalp sensor array with 56 triaxial OP-MEG sensors. (C) The chest sensor array with 8 triaxial OP-MEG sensors. Retroflective markers for optical motion tracking are attached to both the scalp and chest scanner casts.

An on-scalp sensor array with 56 triaxial OP-MEG sensors (168 channels, i.e., oriented axes) (Neuro1 system, QuSpin Inc., Louisville, CO, USA) was mounted in a participant-specific 3D-printed head cast designed based on the participant’s structural MRI scan (Chalk Studios, London, UK). Eight additional triaxial OP-MEG sensors (24 channels, i.e., oriented axes) mounted in a 3D-printed scanner cast were fixed to the front of participant’s chest, at the level of the heart (see Fig. 2). It should be noted that, in the present study, the chest sensor array was included primarily to provide a proof of concept for future, more detailed, magnetocardiographic recordings using OP-MEG sensors. In the present analysis, data from the chest array were used solely to obtain a heartbeat signal for R-peak extraction (see Section 3.2.7), such that additional ECG hardware was not required.

All data were recorded inside a magnetically shielded room (MSR) at University College London, with internal dimensions of 438 cm x 338 cm x 218 cm (Magnetic Shields Ltd, Staplehurst, UK), constructed from two inner layers of 1 mm mu-metal, a 6 mm copper layer, and then two external layers of 1.5 mm mu-metal. Degaussing was applied prior to each scanning session to obtain magnetic equilibrium inside the MSR (Altarev et al., 2015). In addition, automated sensor-level field nulling was performed using the inbuilt field cancellation coils to further reduce residual fields around the OP-MEG vapour cell prior to acquisition (Osborne et al., 2018; Tierney et al., 2019). The data were subsequently recorded in open-loop mode and sampled at 1,500 Hz.

#### 3.2.3 Paradigm

A total of four runs of data acquisition were conducted. During all runs, the participant was seated on a non-magnetic chair in the middle of the MSR.

The first two runs were eyes-open seated recordings of each 10-minute duration, hereafter referred to as silent recordings. In the first run, the participant was instructed to sit as still as possible, while looking straight ahead. In the second run, the participant was instructed to change the position of their head intermittently (approximately 2–3 times per minute) by rotating their head into any direction they wished, but remain still otherwise.

In the second half of the experiment, an auditory evoked-response paradigm, adapted from Garrido et al. (2008) was used. Tone duration was set to 70 ms (including 5 ms rise and fall times) and a frequency of 500–800 Hz in steps of 50 Hz. An auditory tone of the same frequency was presented 1–11 times before randomly switching to an auditory tone of a different frequency. The inter-stimulus interval was set to 0.9 seconds (no jitter). Stimuli were delivered via PsychoPy (Peirce, 2009) and the sound was played via MEG-compatible ear tubes with transducers (Etymotic; Lucid Hearing Holding Company, LLC, TX, USA) connected to a speaker outside the magnetically shielded room. The volume was adjusted to a comfortable level as specified by the participant. During the third run, the participant was instructed to sit as still as possible and face straight ahead, while 548 auditory stimuli were delivered during a recording duration of 8 minutes and 30 seconds. During the fourth run, the participants were instructed to rotate their head intermittently to a freely chosen new angle (2–3 times per minute) and remain as still as possible in between rotations. In this final run, 577 auditory stimuli were delivered during a recording duration of 9 minutes and 20 seconds.

#### 3.2.4 Head and torso position tracking

The head and torso positions were tracked optically throughout the experiment by using an OptiTrack motion capture system (NaturalPoint Inc., Corvallis, OR, USA).

Four retro-reflective markers were attached to the head scanner cast and four additional retro-reflective markers were attached to the chest scanner cast.

The retro-reflective markers were tracked passively at 120 Hz by six OptiTrack Flex13 motion capture cameras, placed in the four corners of the room (four cameras in the front two corners with one camera high up and one low down, and two cameras in the back two corners at medium wall height) (see Seymour et al., 2021).

The optical tracking data were processed in OptiTrack’s proprietary motion capture software Motive 3.1 (NaturalPoint Inc., Corvallis, OR, USA). In Motive, based on the joint translation of the retro-reflective markers, two rigid bodies were defined, providing a measure of the position and rotation of head and chest over time. Prior to solving the rigid bodies, the marker trajectories were visually inspected, gaps were interpolated, and a bi-directional 4-th order Butterworth 2 Hz low-pass filter was applied to exclude high-frequency noise due to vibrations or spurious errors (Seymour et al., 2022).

Then, the movement trajectories of the solved rigid bodies were temporally aligned with the OP-MEG data using a 5 V trigger pulse recorded in the OP-MEG data, which marked the start and end of the OptiTrack recording. Based on this trigger pulse, the two datasets were trimmed to their common recording window, and the motion-tracking data were resampled to match the sampling rate of the OP-MEG data. Finally, the positions and orientations of the OP-MEG sensors were derived for each frame by transforming the rigid body movement trajectories into sensor-specific 6-degree of freedom position and orientation timeseries using permuted Kabsch alignment (Kabsch, 1976) (for details about the procedure, see Mellor et al., 2023).

#### 3.2.5 Co-registration

At the end of the recording session, an optical scan of the participant’s upper body was obtained using a handheld Einscan H 3D scanner (Shining 3D, Hangzhou, China; https://www.einscan.com/handheld-3d-scanner/einscan-h/). During the optical scanning, the participant remained seated, facing forward, in the same position as during the experiment, and was still wearing the head and chest OP-MEG scanner casts.

**Fig. 3.**
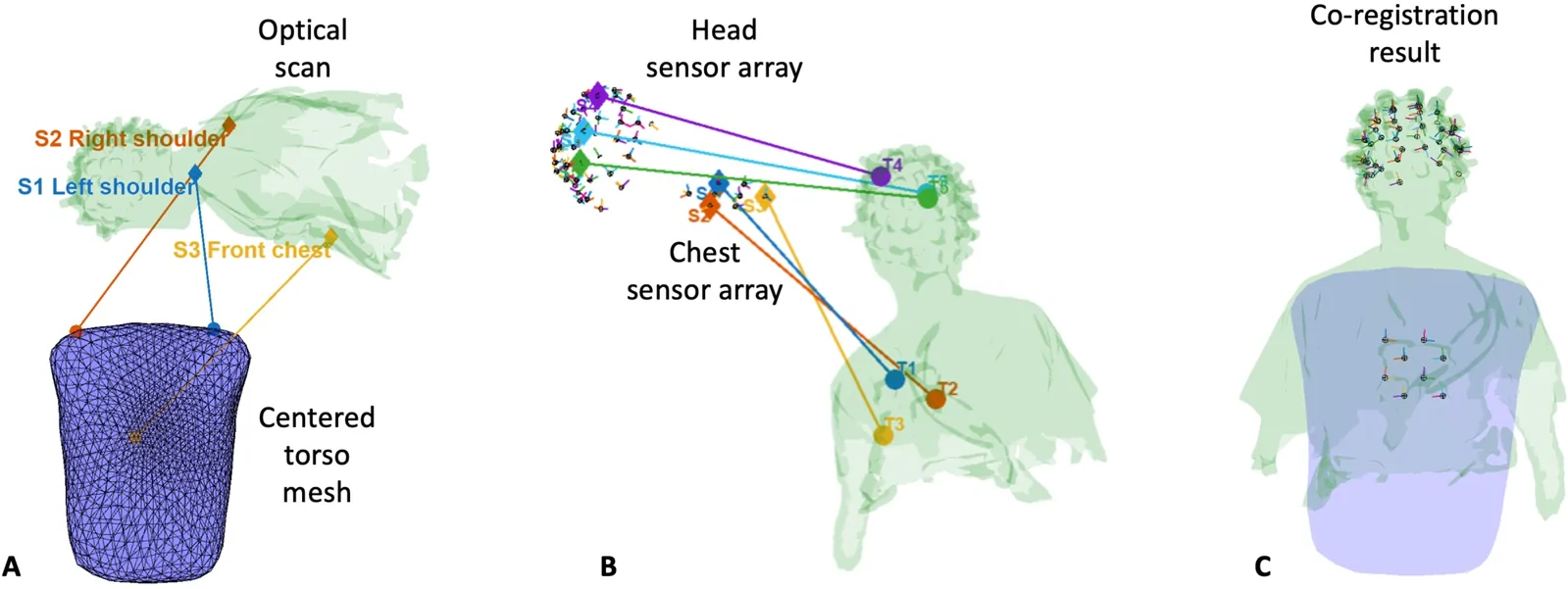
Alignment of the optical scan of the participant’s torso, head, and chest OP-MEG sensor arrays, and the volume conductor torso mesh. (A) Alignment of the optical scan to the centred torso mesh using anatomical landmarks. (B) Alignment of the heart and brain sensor arrays to the aligned optical scan using selected sensor locations. (C) The final integrated result, with both sensor arrays positioned relative to the torso mesh after sequential rigid-body alignment. In panels (A) and (B), the source landmarks and corresponding target landmarks are indicated to illustrate the alignment procedure.

The optical scan of the torso was used to co-register the volume conductor model of the torso and the OP-MEG sensor locations in analysis space. For this purpose, first the anatomical meshes of the heart, lungs, and torso were centred in 3D space. Then, in the optical scan of the torso, two sets of fiducial points were manually annotated. The first set of fiducials was used to spatially align the 3D scan with the anatomical mesh of the torso. Specifically, three fiducial points—two on the shoulders and one in the centre of the torso—were identified on both the optical scan and the torso mesh. These points were then used to compute the rigid body transformation that aligned the optical scan to the torso mesh coordinates, using the least-squares solution based on singular value decomposition. The second set of fiducials was used to spatially align the OP-MEG sensor coordinates with the torso mesh. Specifically, the 3D coordinates of the OP-MEG sensors, above which retroflective markers had been attached, were annotated in the optical scan (now aligned with the volume conductor model of the torso). The locations of the corresponding sensors were extracted from the SPM M/EEG data object. These points were used to compute the rigid body transformation that aligned the OP-MEG sensor coordinates with the corresponding sensor position in the optical scan. Since the optical scan had previously been aligned with the torso mesh, the OP-MEG sensor coordinates became simultaneously aligned with the anatomical mesh of the torso. The co-registration process is illustrated in Figure 3.

#### 3.2.6 OP-MEG data pre-processing

The OP-MEG data were loaded into SPM format generating an SPM M/EEG data object, hereafter referred to as the dataset. First, bad channels with clear deviation from the median power for frequencies up to 100 Hz were rejected based on visual inspection of the power spectral density. This led to the rejection of a total of 46 channels from 18 sensors (i.e., since the triaxial OP-MEG sensors have three independent channels, it is possible to perform rejection channel-by-channel). We inspected all four runs and rejected the same set of bad channels across all runs.

Next, the data were split into two separate datasets—one containing the channels from all sensors in the head scanner cast, and a second containing all channels from the sensors in the chest scanner cast. Following this, both datasets were high-pass filtered at 2 Hz and low-pass filtered at 70 Hz to remove low and high frequency noise and band-stop filtered between 48 and 52 Hz to remove line noise. All filters were 5-th order bi-directional Butterworth filters as implemented in *spm_eeg_filter* (Litvak et al., 2011).

#### 3.2.7 Heartbeat extraction from chest OP-MEG sensors

The R-peak, which occurs during ventricular depolarization, represents the moment of largest electrical current flow in the cardiac cycle, making it a reliable marker for identifying the timing of heartbeats in cardiac source imaging (i.e., ECG and MCG recordings) (Hall & Hall, 2021). To identify the R-peaks in the present study, the data from the chest-mounted OP-MEG sensors were visually inspected, and the channel most-closely resembling an ECG waveform was selected. R-peaks were then extracted, using the Matlab function *findpeaks*, allowing for a minimum peak distance of half a second, corresponding to a maximum heart rate of 120 BPM. R-peaks outside the 5th to 95th percentile of the amplitude range were defined as outliers and excluded.

#### 3.2.8 Empirical CDM estimation

For the empirical data, we derived four CDM estimates (one per recording), following the methodology described above. For each recording, the detected R-peaks were split into training trial set and a test trial set. The training trials were used to estimate the CDM. CFA-correction performance was evaluated using the test trials.

We employed a model of the heart with two dipoles, given that—in absence of an anatomical MRI scan—we had no knowledge about the true location of the participant’s heart. Based on our simulation results, the two-dipole model would largely compensate for the location error and provide an accurate characterization of the heart’s magnetic field.

To systematically select the 2 dipole locations, 2 randomly oriented unit vectors were generated and scaled by 2 different magnitudes randomly drawn from 20 values, linearly spaced between 10 and 50 mm. These vectors pointed outward from the centre of the heart mesh of the volume conductor model, with their end points indicating the chosen locations of the two dipoles. This was done once, and the two selected dipole locations were then kept constant throughout training and test trials.

For each training trial, a data window from 400 ms before to 600 ms after the R-peak was extracted to cover the full cardiac cycle. To factor out amplitude variations between cardiac cycles, we divided each training trial’s data by the R-peak amplitude of that trial, as measured by the chest OP-MEG channel.

The training data from all trials were concatenated into a single training data matrix, and the lead field matrices from all head rotations (calculated at the time point of the R-peak within each trial) were concatenated in the same order as the training data, resulting in a single lead field matrix. Using the concatenated training data and lead field matrices, the least-squares estimate of the CDM was obtained via the Moore–Penrose pseudoinverse according to Equation (4).

#### 3.2.9 Assessment of CFA-correction performance for the silent condition

We first assessed CFA-correction performance for the two silent recordings. This yielded 367 training and 367 test trials for the recording during which the participant had remained facing forward, and 366 training and 367 test trials for the recording during which the participant had intermittently rotated their head.

After deriving the CDM estimate using the training trials, we used the CDM estimate to correct the individual test trials. Specifically, for each trial, we extracted a data window of equal length as the CDM estimate (i.e., 400 ms prior to 600 ms post R-peak) and computed the corresponding lead field matrix using the OP-MEG channel orientations and positions at the time of the R-peak. We then scaled the CDM estimate to the R-peak amplitude of the to-be-corrected trial by multiplying all data points in the CDM estimate with the amplitude of the R-peak as measured by the chest OP-MEG channel. Finally, the CDM estimate was projected through the lead field matrix to generate a prediction of the CFA for the current trial. This CFA prediction was then subtracted from the windowed trial data, as described in Equation (6), yielding the CFA-corrected trial data.

To assess CFA-correction performance, we computed the correlation coefficient between the CFA prediction and the uncorrected data at time of the R-peak.

#### 3.2.10 Assessment of CFA-correction performance for the condition with auditory stimulus

Next, we tested the CFA-correction performance using the data from the two recordings with the auditory evoked-response paradigm. We focused this analysis on the detection of the N100, which is a well-researched early deflection, occurring usually between 80 and 120 ms after tone onset (Näätänen & Picton, 1987). Given that, in our study, the sound was played from a speaker outside the magnetically shielded room and travelled via a 5-metre long silicone tube before reaching the participant’s ears, we corrected the stimulus timings by +30 ms to account for both system latencies (e.g., audio buffering and hardware delays) and the fact that sound travels approximately 330 m/s in air at room temperature (Cramer, 1993).

For both recordings with the auditory stimulus, R-peaks were assigned to the training set if no auditory stimulus occurred from 350 ms before to 150 ms after the R-peak (i.e., to avoid overlap of the R-peaks with any of the major auditory-evoked field components).

To generate the test set, the auditory stimuli were sorted based on their proximity to the nearest heartbeat, and all auditory stimuli with an R-peak broadly overlapping the N100 window, that is, in a window 50–150 ms after the auditory stimulus, were assigned to the test set. Auditory stimuli close to an outlier R-peak, that is, in a window 200 ms before to 200 ms after the stimulus, were excluded from the analysis, to avoid including corrupted data at this stage. This exclusion range was chosen because, at a regular resting heart rate, the full period of cardiac activity from atrial depolarization to ventricular repolarization is approximately 500–600 ms and the typical time from ventricular depolarization to ventricular repolarization is approximately 350 ms (Hall & Hall, 2021).

This procedure yielded 270 training and 65 test trials for the recording during which the participant had remained facing forward, and 314 training and 80 test trials for the recording with intermittent head rotation.

To derive the CDM estimate, we followed the same procedure used for the silent recordings, with one addition: prior to applying the CFA correction, the CDM estimate was aligned to the heartbeat nearest to the auditory stimulus by matching the R-peaks and shifting all time points in the CDM estimate moment vector accordingly. If, after alignment, the CDM estimate was shorter than the test signal, the moment vectors were zero padded to match the test signal length.

To derive an estimate of the ground-truth N100, for both recordings we selected the CFA-free trials, defined as all trials without any R-peak in a window range from 200 ms pre-stimulus to 200 ms post-stimulus. This yielded 264 ground-truth trials for the facing-forward dataset and 273 ground-truth trials for the head-rotation dataset.

Ground-truth, uncorrected test, and corrected test data were epoched relative to the auditory trigger and baseline corrected using a 100 ms pre-stimulus interval. For each trial, the N100 response was quantified by averaging the signal in the window 80–120 ms post-stimulus, yielding a single value per sensor.

CFA-correction performance was evaluated using a paired nonparametric bootstrap approach with 20,000 resamples. On each iteration, trials were resampled with replacement, using the same trial indices across uncorrected and corrected datasets to preserve the paired nature of the methods comparison.

For each bootstrap sample, the uncorrected and corrected N100 sensor-level data were compared with the ground truth using RMSE, Pearson correlation (*r*), and absolute bias, defined as the absolute value of the mean difference from the ground truth. We included these three metrics to capture complementary aspects of correction performance: RMSE captures overall error magnitude; Pearson correlation captures spatial similarity in sensor topographies, and absolute bias captures systematic over- or underestimation. For each metric, we report the mean of the bootstrap distribution and the 95% confidence interval (CI).

After sensor-space analysis, the data were projected to source space. The head model was constructed by warping the canonical SPM template mesh into the native space of the participant’s structural MRI scan, and the forward model was computed based on the inner skull surface using the corrected-sphere single-shell method as implemented in SPM (Litvak et al., 2011; Nolte, 2003). Source reconstruction was then performed in SPM25 using the distributed minimum-norm solution with an independent and identically distributed (IID) source prior covariance to localize the N100 response within the 80–120 ms post-stimulus window and 1–48 Hz frequency range. Model fit was assessed using the percentage of variance in the sensor-level data explained by the reconstructed source-level estimates, alongside visual inspection of cortical surface maps of the reconstructed source activity within the N100 time window.

#### 3.2.11 Performance relative to established artefact correction methods

Finally, we compared the effectiveness of CFA correction via CDM estimate with that of established artefact correction methods. As alternative methods we chose ICA, which is widely used for CFA correction in EEG, MEG, as well as OP-MEG recordings (Seymour et al., 2022); and homogeneous field correction (HFC; Tierney et al., 2021), which is an artefact correction method specifically developed for OP-MEG that approximates magnetic interference as a spatially constant field.

We employed the popular fastICA algorithm as implemented in the Fieldtrip function *ft_componentanalysis* (Oostenveld et al., 2011). The topographies and time courses of all components were manually inspected to identify components relating to the heartbeat. For confirmation, we computed the frequency–domain coherence between each component and the OP-MEG channel previously identified as most clearly resembling an ECG signal. Coherence was estimated from 0 to 100 Hz using multi-taper Fourier transform as implemented in the Fieldtrip functions *ft_freqanalysis* and *ft_connectivityanalysis* (Oostenveld et al., 2011). HFC was implemented via SPM’s *spm_opm_hfc* function (Litvak et al., 2011) using default settings.

In addition to employing the individual correction methods in isolation, we also evaluate potential synergies between correction methods. Specifically, we tested whether application of HFC after either CDM or ICA would improve results, since both CDM and ICA specifically target the CFA, whereas HFC reduces general interference from outside the head and promises to be especially beneficial for recordings with participant movement (Tierney et al., 2021).

### 3.3 Software

All analyses were conducted in MATLAB R2025b (Mathworks Inc., Natick, MA, USA) using the Statistical Parametric Mapping (SPM25; Litvak et al., 2011) and FieldTrip (Oostenveld et al., 2011) toolboxes, and custom scripts archived on Zenodo (https://zenodo.org/records/20209782). The corresponding dataset can be accessed on Zenodo (https://zenodo.org/records/20202674).

## 4 Results

In this section, we first present the simulation results, which provide the primary validation of the proposed model-based CFA-correction approach. We then present an empirical proof-of-principle demonstration of the full pipeline under realistic recording conditions.

### 4.1 Simulations: Results

The simulations were designed to validate the model-based CFA correction approach under controlled conditions. Specifically, we examined the projection of the cardiac field across varying head rotations and assessed the effectiveness of CDM-based CFA correction under different modelling assumptions and sources of error.

#### 4.1.1 Simulation of the heart’s electromagnetic activity

**Fig. 4.**
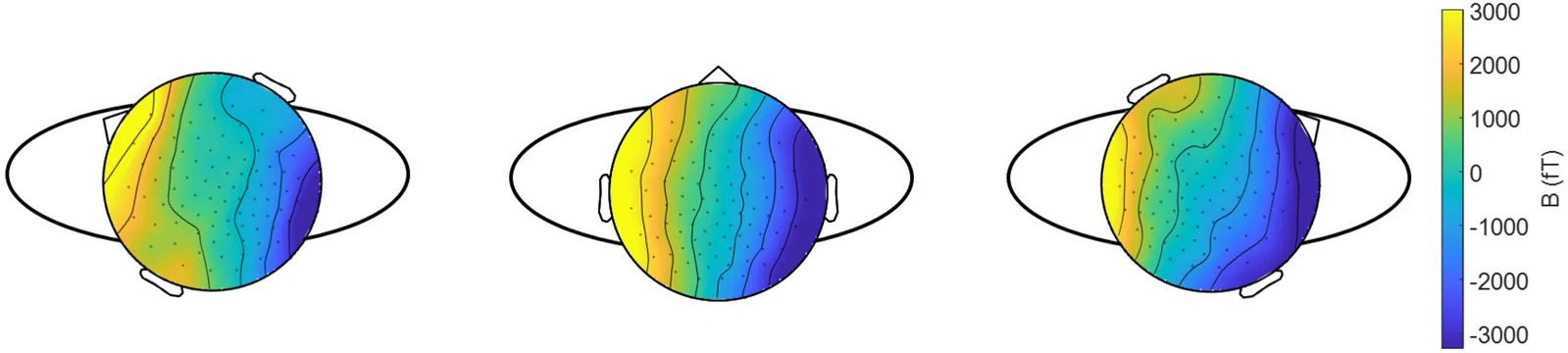
2D topographical field maps of simulated cardiac activity, measured by an OP-MEG sensor array on the scalp. All plots show the radial channel readings at time of the simulated R-wave. Left: Head rotated 60˚ to the left. Middle: Head facing straight ahead. Right: Head rotated 60˚ to the right. Ellipses underneath the topographical representations indicate the relative rotation of the torso.

Figure 4 shows example data for head rotation angles of -60˚, 0˚, and 60˚ relative to the torso. Note that the cardiac field at the scalp level remains aligned to the torso, instead of rotating with the head.

#### 4.1.2 Accuracy of the CDM estimate

Simulations confirmed that the CDM estimated in a given head rotation can be used successfully to correct an unseen test signal (i.e., a CFA recorded in a different head rotation). We tested this hypothesis by estimating the CDM from simulated training data where the head was turned 60˚ to the left and subsequently using this CDM estimate to correct the CFA in simulated test data where the head was at 0˚ (i.e., facing forward). Even in this simplified case, in which the CDM was estimated with training data from a single head rotation, most of the CFA was successfully removed. Before correction, the average CFA amplitude across channels was approximately 22 times larger than the reference signal. After CFA correction, the residual artefact amounted to only 9% of the reference signal (see Supplementary Material 2).

**Fig. 5.**
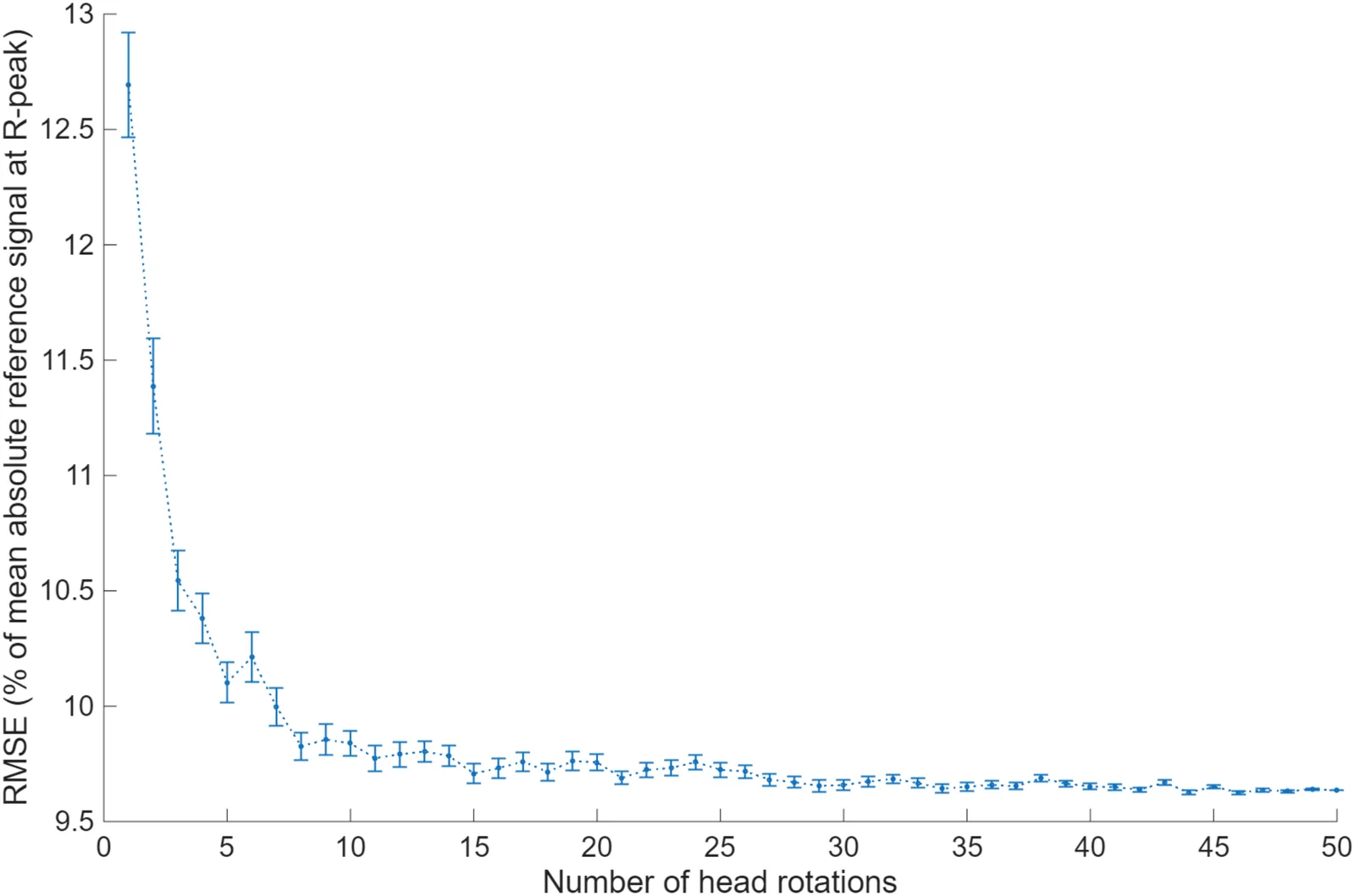
Channel-level CFA-correction error (RMSE) for CDM estimates with data from an increasing number of head rotations. With an increasing number of head rotations, the accuracy of the CDM estimate gradually improves. For each number of head rotations, RMSE is averaged across 100 runs, each using a different random sample of head-rotation angles. RMSE is expressed as a percentage of the reference signal, which was scaled to 10% of the peak CFA amplitude to match the amplitude of a typical evoked field in the brain. Whiskers represent standard errors of the average RMSE across runs.

Simulations furthermore confirmed that CDM estimation accuracy increased when a greater number of head rotations were used for CDM estimation. As shown in Figure 5, the most significant reductions in RMSE between the corrected signal and the reference signal were achieved when training data from up to 10 different head rotations were included in the CDM estimate. After that, including data from more head rotations yielded marginal returns, and the error stabilized at around 9.7% of the amplitude of the reference signal.

#### 4.1.3 Recovery of brain activity time locked to the cardiac cycle

In the next set of simulations, we tested the CDM estimation accuracy using a simulated ERF at the location of the right insula. Specifically, we estimated the CDM with simulated training data from 20 distinct head rotations, and used this CDM estimate to correct unseen simulated test data (facing forward) where the insula ERF had been obscured by the CFA.

**Fig. 6.**
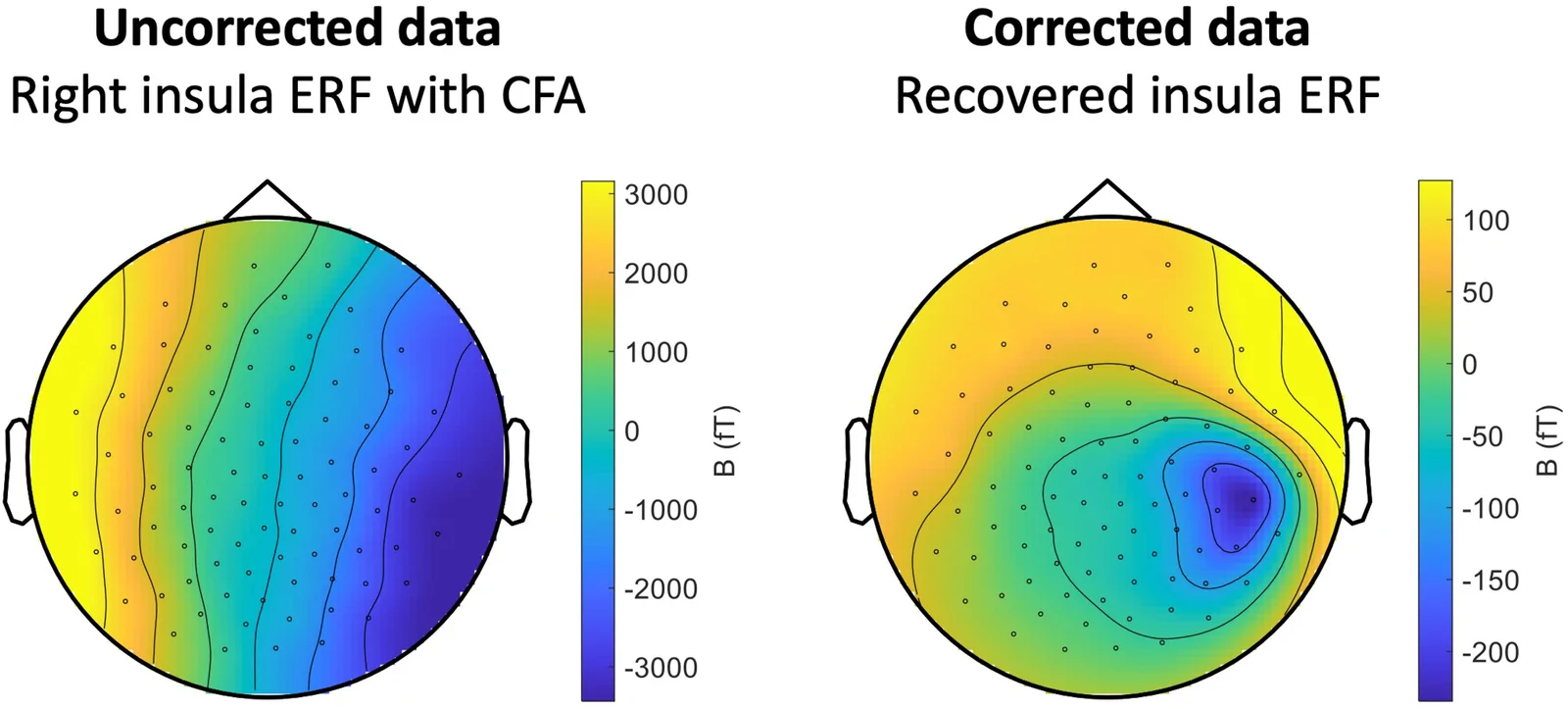
2D topographical field maps of radial channel measurements of an ERF simulated at the location of the right insula before and after CFA correction. Left: insula simulation with CFA. Right: insula simulation after CFA correction. The CDM used for correction was estimated with data from six distinct head rotation angles.

Simulation results showed that CFA correction successfully recovered the original spatial pattern of the insula signal, as measured at channel level (Fig. 6). Before correction, the simulated CFA as measured by the OP-MEG channels was approximately five times larger in amplitude than the simulated brain signal. After correction, the residual artefact was reduced to 1.46% of the simulated brain signal.

#### 4.1.4 Robustness of the CDM estimate to cardiac source misplacement in the forward model

In the next set of simulations, we assessed the impact of mis-modelling the heart source location in the forward model. Specifically, we tested whether CDM estimation accuracy would decrease when the dipole used to model the heart’s electromagnetic activity was placed at increasing distance from the true heart location.

**Fig. 7.**
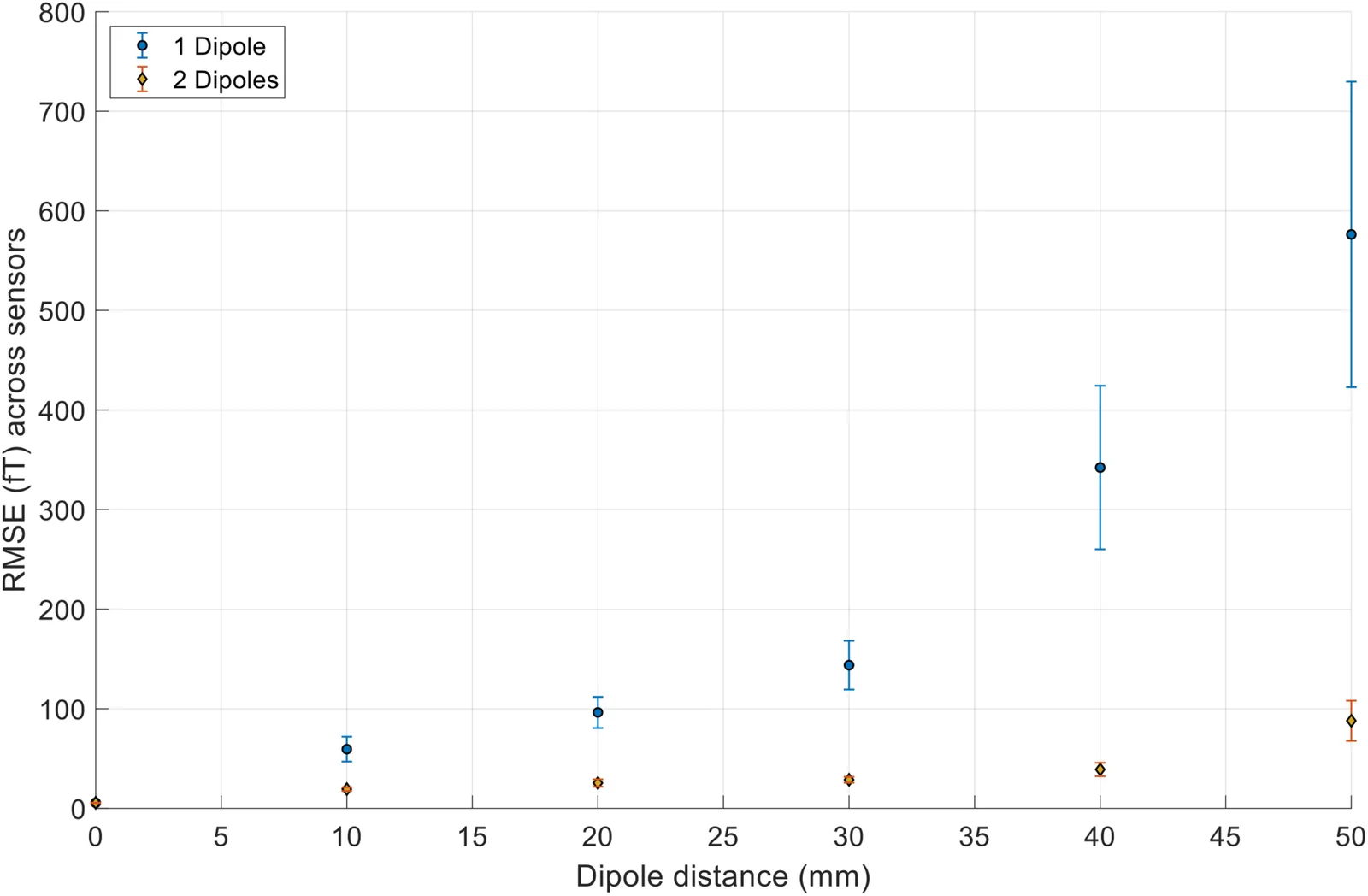
Error (RMSE) between the corrected signal and the reference signal for a CDM estimate with a single dipole, compared with a CDM estimate with two dipoles. Error values are displayed as a function of increasing dipole distance from the true heart location. For each distance, 10 runs with random dipole orientations were performed and the resulting RMSE values were averaged. Whiskers represent standard errors of the average RMSE across runs.

The results from these simulations showed that CDM estimation accuracy, measured by the RMSE between the corrected data and the reference signal, decreased with increasing distance from the true heart location (see Fig. 7). This was the case both when the CDM was estimated with a single dipole and when the CDM was estimated with two dipoles, with both models showing a similar strength of correlation between RMSE and dipole distance from the true heart location (*r* = .92 and *r* = .89, respectively). However, their rate of change differed significantly. For the model with a single dipole, the maximum RMSE increase at a distance of 50 mm was more than 100-fold, from 5.6 to 567.34 fT. In contrast, for the model with two dipoles, the maximum RMSE at 50 mm distance was only 87.95 fT.

#### 4.1.5 Evaluation of the validity of a standardized torso volume conductor model

**Fig. 8.**
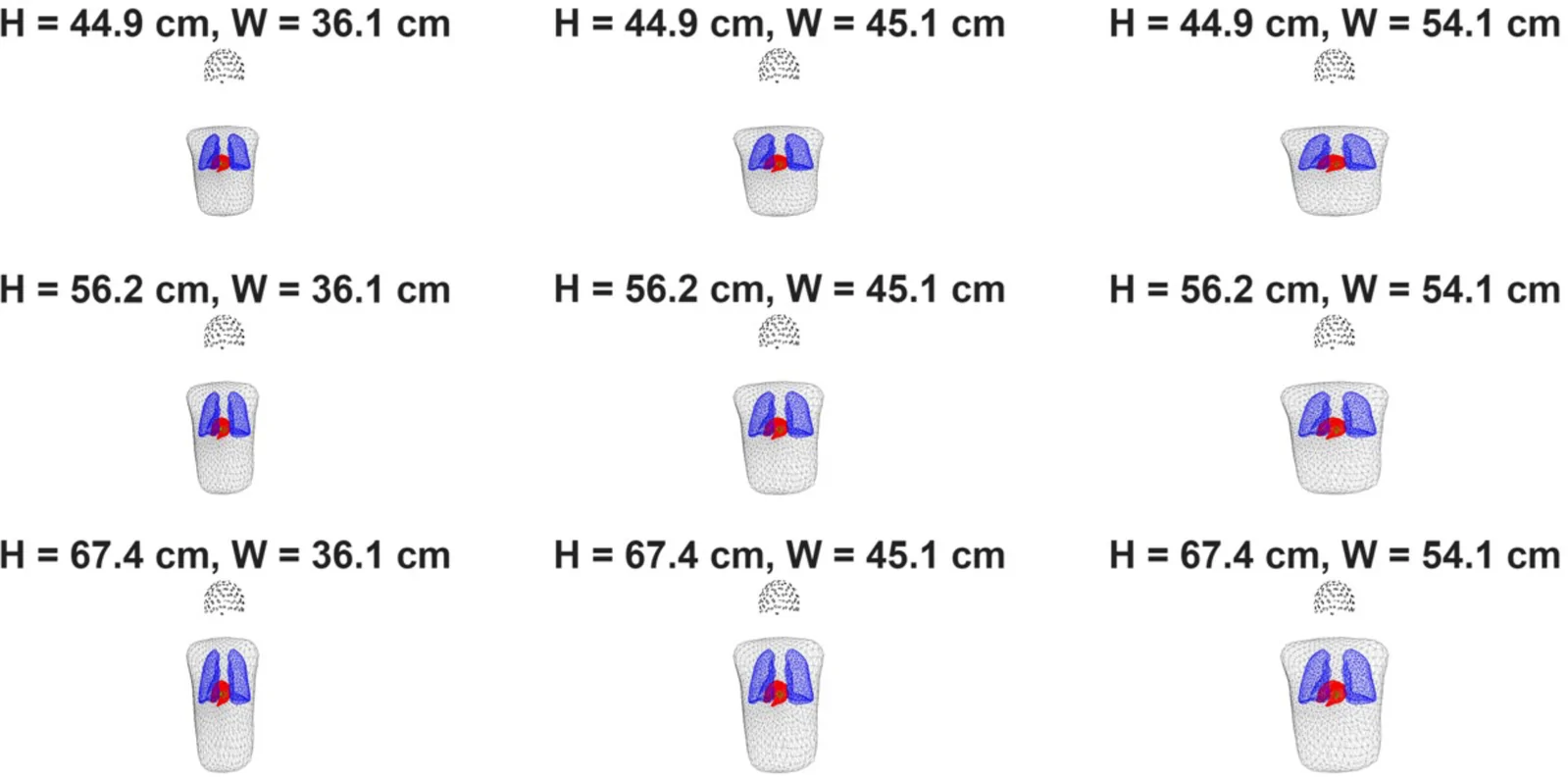
Tested torso proportion geometries. The original mesh (centre) was scaled in height and transverse dimensions by ±20% to derive a total of nine distinct physiques.

In the final set of simulations, we examined the effect of employing a standardized volume conductor model for the torso, instead of a personalized torso model. For this purpose, we simulated eight additional torso geometries (Fig. 8) and evaluated whether CDM estimates derived with these torso models decreased CFA correction accuracy.

**Fig. 9.**
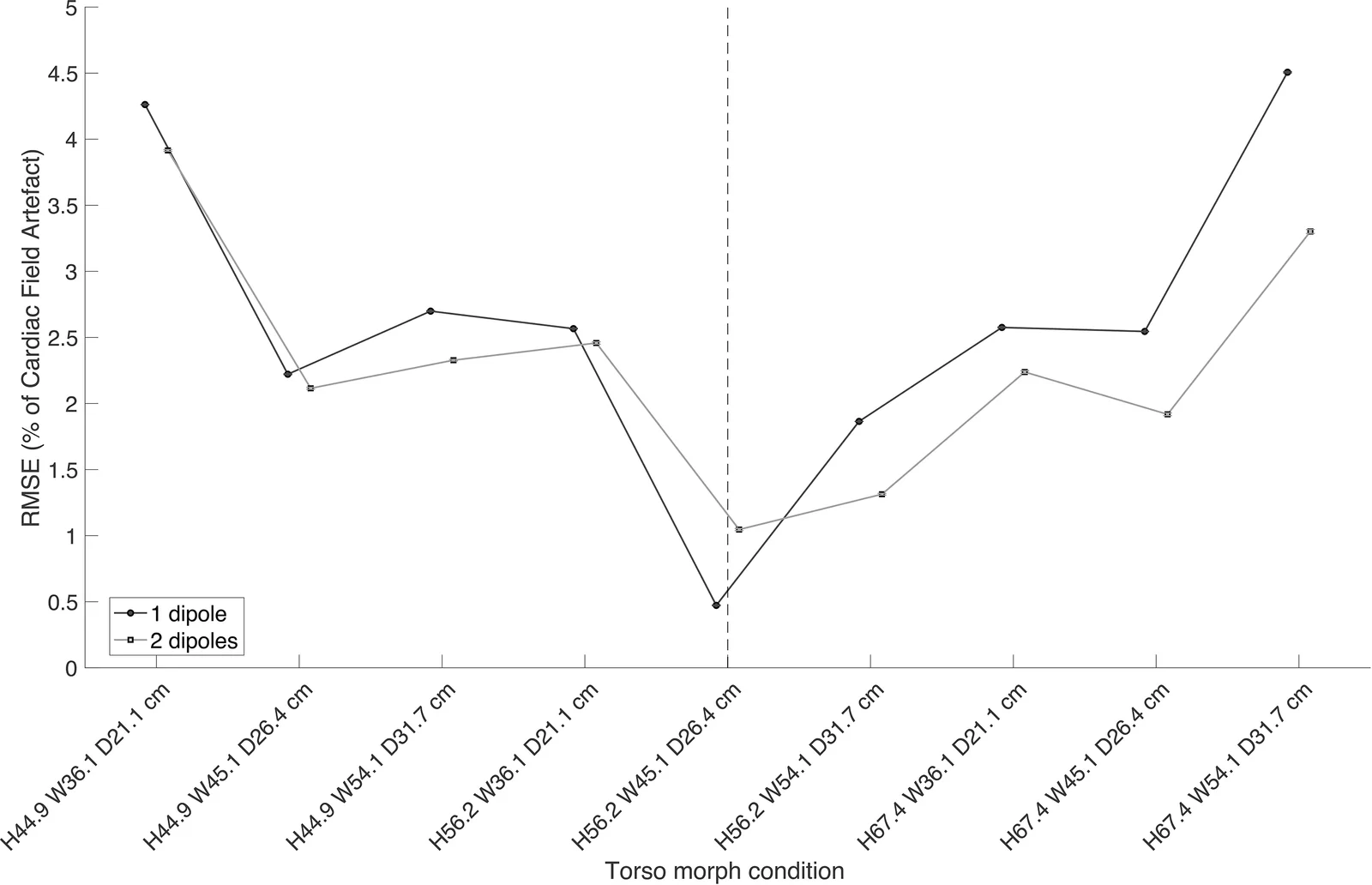
Simulation results: CDM estimation errors as a function of error in torso model. Estimation errors (RMSE) are shown as a percentage of the simulated CFA and were computed on a sensor-by-sensor basis relative to each sensor’s own test-signal amplitude. The vertical line in the centre indicates the body proportions corresponding to the original torso model, which was used to generate the test data. Whiskers represent standard errors of the average RMSE across runs.

Results indicated that, when the CDM was estimated using any of the morphed torso volume conductor geometries and applied to correct test data generated with the original unscaled torso model, CFA correction performance—quantified as the RMSE between the corrected signal and the reference signal at time of the R-peak—remained within 4.04% of the accuracy obtained with a CDM estimated using the original torso mesh (Fig. 9). Specifically, for the one-dipole model, the lowest RMSE, obtained with the original torso mesh, amounted to 0.47% for the CDM estimated with a single dipole and to 1.05% for the CDM estimated with two dipoles, whereas the maximal RMSE with the most strongly morphed torso geometries amounted to 4.51% for the CDM estimated with a single dipole and to 3.92% for the CDM estimated with two dipoles.

### 4.2 Empirical measurement: Results

#### 4.2.1 CFA-correction performance for the silent condition

**Fig. 10.**
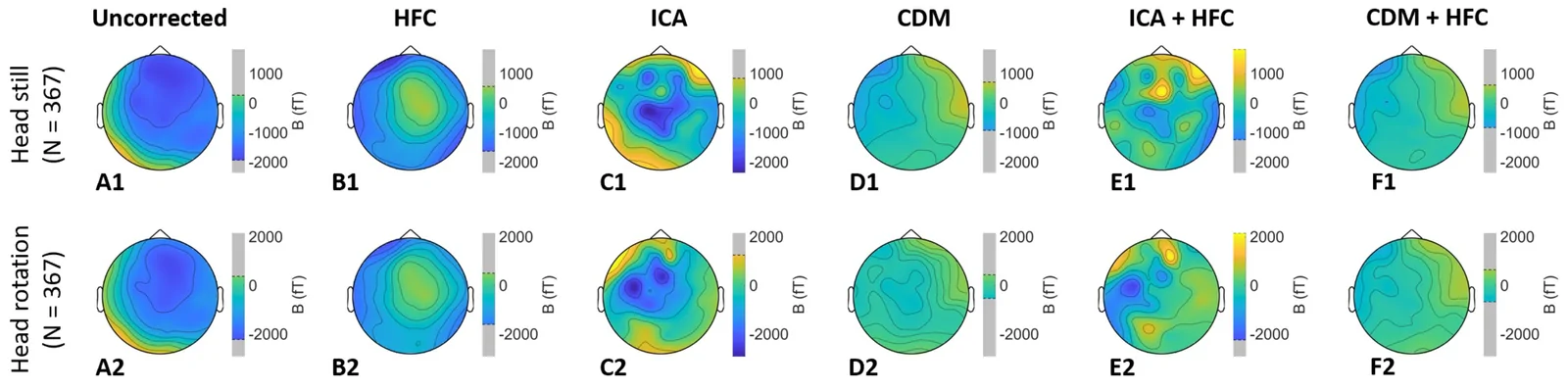
2D field maps of data from the rest condition trial averaged to the time of the R-peak. Top row: recording with the participant facing forward; bottom row: recording with intermittent head rotation. From left to right: (A) uncorrected data with cardiac artefact; (B) homogeneous field correction (HFC); (C) independent component analysis (ICA); (D) cardiac dipole moment estimate (CDM); (E) ICA followed by HFC; (F) CDM followed by HFC. For each recording, colorbar scale ranges are shared across the 2D field maps to facilitate visual comparison; for each plot, colorbar values outside the signal range are indicated by vertical hatching, with limits defined by the maximum sensor amplitudes. Displayed data are from channels oriented radially to the scalp only.

We started the empirical data analysis by assessing the two silent recordings. This analysis confirmed that the CFA featured prominently when the data were trial averaged to the R-peak. As shown in Figure 10, the observed field pattern corresponded to that of the simulated data, with a maximum amplitude measured above the left lateral regions, and a gradual decrease in field strength toward the right fronto-lateral regions. Across channels, the average measured CFA amplitude was 1212 fT (*SD* = 814) in the recording where the participant faced forward (Fig. 10, A1) and 1349 fT (*SD* = 889) in the recording during which the participant intermittently rotated their head (Fig. 10, A2).

After CFA correction using the CDM estimate, the average measured amplitude at time of the R-peak decreased to 242 fT (*SD* = 182 fT) in the recording where the participants faced forward (Fig. 10, D1) and 301 fT (*SD* = 193) in the recording during which the participant intermittently rotated their head (Fig. 10, D2). For both recordings, the measured field after correction exhibited a markedly more uniform amplitude distribution across the scalp.

These results compared favourably against correction with HFC or ICA. Compared with correction with HFC (Fig. 10, B1/B2), correction via the CDM estimate reduced the R-peak amplitude by 51.7% for the facing-front recording and 38.5% for the recording with intermittent head rotation. Compared with correction with ICA (Fig. 10, C1/C2), correction via the CDM estimate reduced the R-peak amplitude by 70.8% for the facing-front recording and 66.1% for the recording with intermittent head rotation.

Best results were obtained by combining the CDM estimate with subsequent HFC correction: relative to CFA correction with CDM estimate alone, for the facing-front recording application of HFC after correction with the CDM estimate yielded a marginal additional reduction in CFA field strength by 1.8%—indicating that most artefact suppression was already achieved by correction with the CDM estimate (Fig. 10, F1). For the recording with intermittent head rotation, application of HFC after correction with the CDM estimate yielded improvement of an additional 14.9% (Fig. 10, F2). In contrast, subsequent HFC substantially improved correction performance after ICA, by 41.3% for the facing-front recording (Fig. 10, E1) and 34.4% for the head-rotation recording (Fig. 10, E2).

On a single-trial level, the CDM estimate explained, on average, 78% (*r* = .88, *SD* = .14) of the sensor-wise variance in raw signal amplitude at the time of the R-peak when the participant remained facing forward, and 71% (*r* = .83, *SD* = .22) when the participant intermittently rotated their head. When the data and CFA predictions were first trial averaged, the explained variance increased to 95% (*r* = .98, *p* < .001) when facing forward and 96% (*r* = .98, *p* < .001) with intermittent head rotation.

Exploratory analysis of factors associated with poorer correction performance during the recording with intermittent head rotation revealed significant negative correlations with both head movement (*r* = -.26, *p* < .001) and total signal power (*r* = -.26, *p* < .001) (see Supplementary Material 3 for details).

#### 4.2.2 CFA-correction performance for trials with auditory stimulus

For the analysis of the recordings with auditory paradigm, we first focused on artefact-free trials to confirm the presence of the expected N100 auditory evoked field (AEF). Artefact-free trials were defined as trials without any R-peak in a window from 200 ms pre-stimulus to 200 ms post-stimulus, because ventricular repolarization at regular resting heart rate completes approximately 300–400 ms post R-peak (Hall & Hall, 2021). This yielded 264 CFA-free trials for the recording during which the participant faced forward and 273 CFA-free trials for the recording during which the participant intermittently rotated their head.

**Fig. 11.**
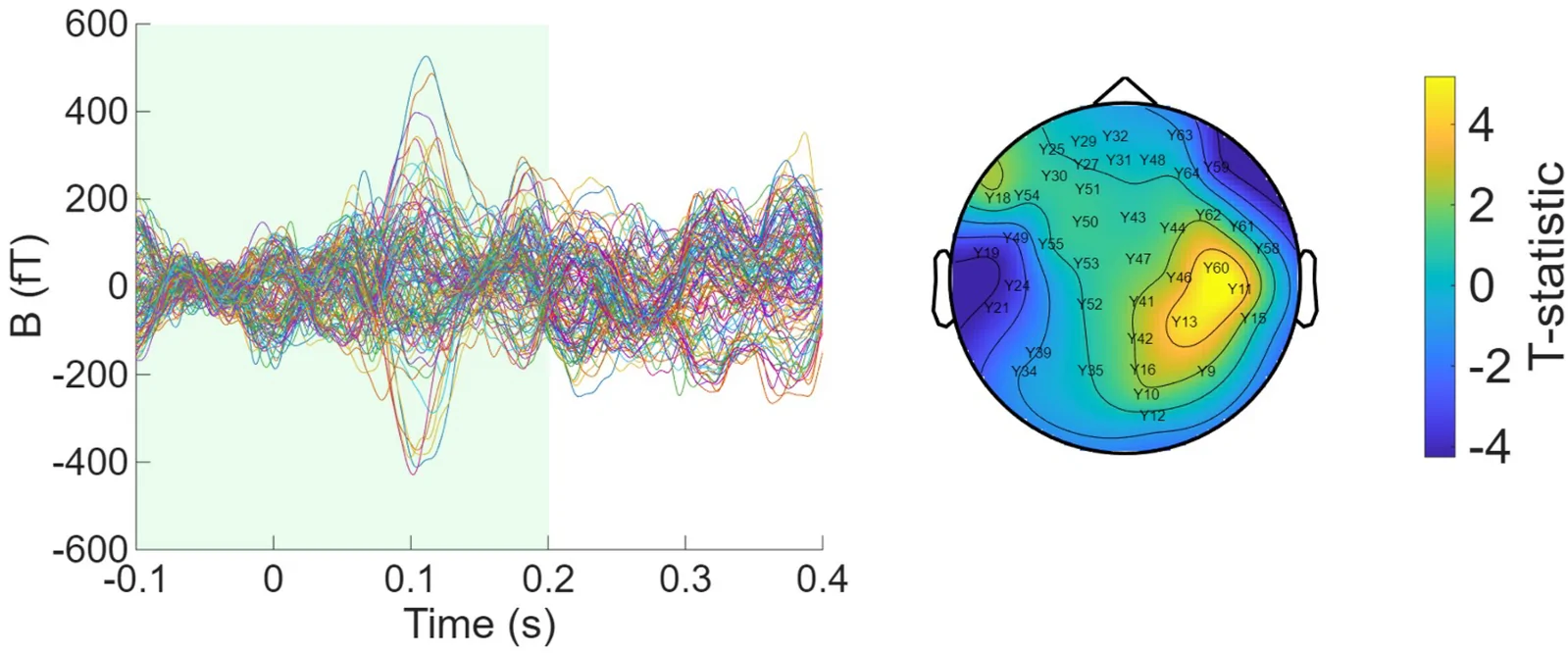
Evoked field (N100) during CFA-free trials (N = 264) of the auditory paradigm (participant facing forward). Channel-level measurements (left) are plotted alongside a 2D field map (right) for data from the typical N100 window (i.e., 80–120 ms). The field map is presented as one-sample t-statistics across trials using baseline-corrected data. The t-values quantify the mean response relative to trial-to-trial variability; positive and negative t-values correspond to response amplitudes above and below the baseline level, respectively. The field map only shows data from channels oriented radially to the scalp. The shaded area indicates the time window used to select artefact-free trials (i.e., peri-stimulus interval without R-peaks).

For both recordings, the CFA-free trials showed clear evidence of an N100 response in the window 80–120 ms post-stimulus, with bilateral sources visible on the field maps (see Fig. 11). For each recording, we used the respective N100 estimate from the CFA-free trials as the ground truth.

**Fig. 12.**
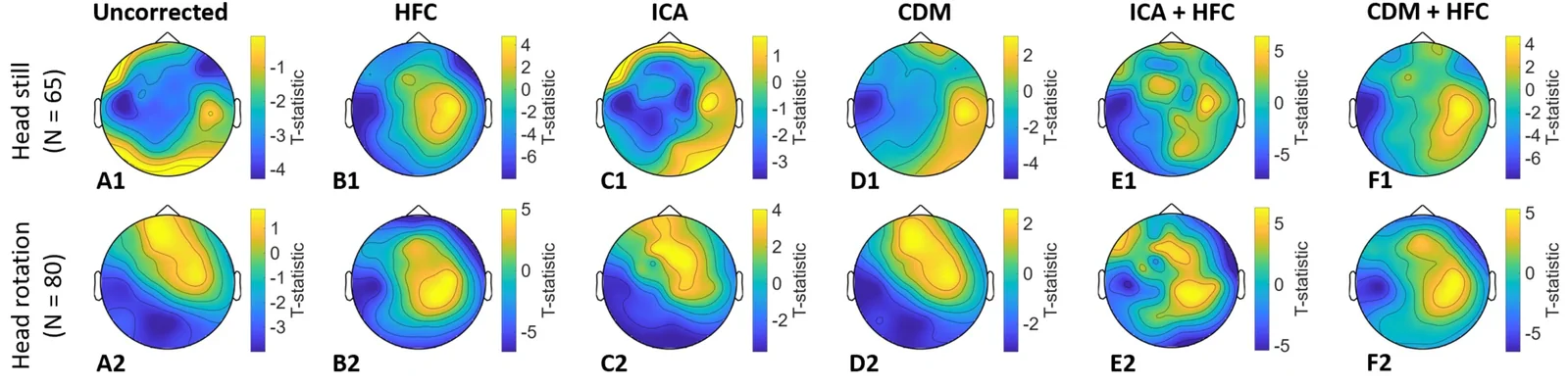
2D field maps of t-statistics for the auditory paradigm before and after CFA correction. Only trials with an R-peak 50–150 ms post-stimulus were included in the test set. Displayed data relate to the expected window range of the N100 evoked response (i.e., 80–120 ms post-stimulus). Top row: data for the recording during which the participant remained facing forward. Bottom row: data for the recording during which the participant intermittently rotated their head. From left to right: (A) uncorrected data with cardiac artefact; (B) homogeneous field correction (HFC); (C) independent component analysis (ICA); (D) cardiac dipole moment estimate (CDM); (E) ICA correction followed by HFC; (F) CDM followed by HFC. The field maps are presented as one-sample t-statistics across trials using baseline-corrected data. The t-values quantify the mean response relative to trial-to-trial variability; positive and negative t-values correspond to response amplitudes above and below the baseline level, respectively. Displayed data are from channels oriented radially to the scalp only.

Figure 12 shows field maps of the averaged auditory evoked response during the facing-forward (top row) and head-rotation (bottom row) recordings, comparing the uncorrected data containing cardiac artefact (panels A) with the results after CFA correction with HFC, ICA, and CDM estimate (panels B–D), as well as combined correction with ICA/CDM followed by HFC (panels E–F).

The CDM estimate explained, on average, 69% (*r* = .82, *SD* = .21) of the raw signal amplitudes when the participant remained facing forward, and 54% (*r* = .74, *SD* = .28) when the participant intermittently rotated their head.

For the test recording, in which the participant remained facing forward, the uncorrected data (Fig. 12, A1) showed poor correspondence with the ground-truth N100 topography at sensor level, with high RMSE, low spatial similarity, and large absolute bias (RMSE = 585.43 fT, 95% CI [367.77, 838.78]; *r* = -.05, 95% CI [-.59, .62]; absolute bias = 231.47 fT, 95% CI [134.22, 332.02]) (see Supplementary Material 4 for full results of all evaluation metrics). All correction methods reduced RMSE and absolute bias relative to the uncorrected data, including CDM alone, but none of the single-method approaches showed a reliable increase in field pattern similarity as the confidence intervals for the bootstrapped correlations between sensor values overlapped zero. The best overall performance was obtained by combined correction with CDM and subsequent HFC (Fig. 12, F1), which yielded the lowest RMSE (156.83 fT, 95% CI [108.53, 229.91]), a significant increase in correlation with the ground-truth N100 field pattern (∆*r* = +.65, 95% CI [+.03, +.98]) and markedly reduced absolute bias (51.69 fT, 95% CI [12.00, 91.80]). ICA in combination with HFC (Fig. 12, E1) was also competitive, yielding the second lowest RMSE (206.41 fT, 95% CI [155.12, 272.00]) and the lowest absolute bias of all tested methods (34.03 fT, 95% CI [2.49, 72.06]). However, CDM followed by HFC achieved lower RMSE (∆ = -49.58 fT, 95% CI [-75.87, -26.66]) and higher correlation with the ground-truth field pattern (∆*r* = +.14, 95% CI [+.07, +.21]). Source estimates indicated a similar directional pattern to that observed for the sensor-level data (see Supplementary Material 5). While visual inspection of source maps indicated some overlap with auditory areas for all solutions, the uncorrected and ICA-only corrected solutions appeared more distributed, which was supported by low explained variance (27.18% and 32.07%, respectively), whereas CDM alone (Fig. 12, D1) showed a clearer N100-like response and intermediate explained variance (45.00%). The HFC-containing solutions showed the strongest source recovery, with highest explained variance for CDM combined with HFC (75.40%), followed by ICA combined with HFC (73.75%), and HFC-only (71.64%).

For the test recording with intermittent head rotation, the uncorrected data appeared less strongly corrupted than in the forward-facing recording (Fig. 12, A2), already showing moderate correspondence with the ground-truth N100 topography at sensor level, although this estimate remained uncertain (RMSE = 491.53 fT, 95% CI [264.11, 816.12]; *r* = .31, 95% CI [-.46, .84]; absolute bias = 196.37 fT, 95% CI [99.85, 291.53]). All correction methods reduced absolute bias relative to the uncorrected data, but only HFC (Fig. 12, B2), ICA followed by HFC (Fig. 12, E2), and CDM followed by HFC (Fig. 12, F2) showed reliable reductions in RMSE. In contrast to the forward-facing recording, none of the correction methods showed a reliable increase in field-pattern similarity relative to the uncorrected data, because the confidence intervals for the bootstrapped correlations all overlapped zero. Among the tested methods, combined correction with CDM followed by HFC again yielded the lowest RMSE (179.71 fT, 95% CI [129.06, 246.51]) together with high correlation with the ground-truth field pattern (*r* = .44, 95% CI [.23, .62]) and low absolute bias (39.48 fT, 95% CI [2.30, 89.42]). Relative to CDM alone (Fig. 12, D2), CDM followed by HFC showed lower RMSE (∆ = -278.44 fT, 95% CI [-588.39, -45.04]) and lower absolute bias (∆ = -81.81 fT, 95% CI [-154.51, -4.74]), and relative to HFC alone it produced a small but reliable reduction in RMSE (∆ = -22.60 fT, 95% CI [-42.61, -5.99]), although differences in correlation and absolute bias were not reliable. ICA followed by HFC also performed well, with low RMSE (215.25 fT, 95% CI [163.92, 281.16]) and the lowest absolute bias of all tested methods (21.19 fT, 95% CI [0.86, 59.09]), but it was not clearly distinguishable from either HFC alone or CDM followed by HFC on the pairwise bootstrap comparisons. Source estimates (see Supplementary Material 5) indicated that the uncorrected data were more strongly corrupted than suggested by sensor-level data alone. Visual inspection of source maps showed that source estimates for the uncorrected and ICA-only solutions were less focal than for the facing-forward recording, which was supported by very low explained variance (1.57% and 11.49%, respectively). The CDM-only solution showed a clearer N100-like response, but with lower explained variance than for the facing-forward recording (20.77%). Again, the HFC-containing solutions showed the strongest source recovery, with the highest explained variance for CDM combined with HFC (70.55%), followed by ICA combined with HFC (67.99%), and HFC-only (62.75%).

## 5 Discussion

The current study introduces a model-based approach for cardiac field artefact (CFA) correction in optically pumped magnetoencephalography (OP-MEG). We show in simulations and an empirical proof-of-principle demonstration that, because participants are free to move their head during OP-MEG acquisition, cardiac source activity can be estimated in a manner that is minimally confounded by concurrent brain activity. By dynamically accounting for the spatial relationship between thorax and scalp, this cardiac dipole moment (CDM) estimate can be used for CFA correction in unseen data from any new head rotation.

Simulations provide the core validation of the method under controlled conditions. Our results demonstrate that the cardiac magnetic field generates a pattern at sensor level that generalizes across head rotations, enabling the creation of a torso-anchored CDM estimate that improves in precision as multiple head rotations are included in the training set. Our simulation results furthermore indicate that the method is relatively robust to inaccuracies in the forward model, making it suitable for application in studies where an anatomical MRI of the participant’s torso is not available.

After confirming the feasibility of the method in simulation data, we collected empirical data on a single subject as a proof-of-principle for the full recording and analysis pipeline, including optical motion tracking. Throughout four recordings, we show that correction with CDM estimate consistently reduces the CFA. In low-noise conditions (silent recordings), it outperformed both independent component analysis (ICA) and homogeneous field correction (HFC). In more realistic recording conditions (auditory response paradigm), best results were obtained when the CDM estimate was combined with subsequent HFC, indicating that the two methods target complementary aspects of signal contamination.

One of the core application areas of model-based CFA correction are studies where the phenomenon of interest is time locked to the cardiac cycle, such as in cardiac interoception research. For the initial validation of the method, it was, however, beneficial to focus on the N100 auditory response as a robust benchmark with well-documented spatial and temporal profile. In contrast, cardiac interoceptive responses such as the HEP are not yet fully characterized in a standardized measurement and analysis framework (Coll et al., 2021; Virjee et al., 2025). In addition, evaluation against established artefact-correction methods would be more challenging for interoceptive responses. For example, ICA is a blind source separation method, which relies exclusively on statistical properties of the data (Stephen, 2019). Therefore, exclusion of the components corresponding to the heartbeat using ICA may simultaneously attenuate or distort brain signal covariant with the heartbeat (Arnau et al., 2023), complicating comparison of correction performance between ICA and other methods. Consequently, usage of an interceptive response for this initial validation study would have introduced additional uncertainty unrelated to CFA correction. We, therefore, view application of the method to interoception studies as a key next step.

An important limitation of the current study is that the empirical proof-of-principle was conducted with a single participant, and that the analysis relied on a standardized volume conductor model for the torso, combined with a relatively simple co-registration procedure. While nearby tissue conductivity inhomogeneities are known to most strongly influence the magnetic field (Mäntynen et al., 2014), the amplitude and spatial distribution of the CFA are nonetheless expected to vary as a function of wider individual anatomical differences. Both our simulations and empirical results indicate that the model is relatively robust to errors in the forward model, including omission of the head, misplacement of the heart dipole, as well as inaccurate torso dimensions—particularly when multiple dipoles are used to model the cardiac source currents. Nevertheless, residual modelling inaccuracies may accumulate and could contribute to the observed ceiling in explained variance in our empirical recordings, which did not exceed 80%. Future studies might, therefore, assess the influence of inter-individual differences in anatomy and physiology on CFA properties and correction performance, and evaluate the benefit of improved co-registration procedures.

A further factor potentially affecting correction performance is movement. During rotation, the head moves through the static field in the room, which is several orders of magnitude larger than the cardiac field, inducing comparatively large signal fluctuations in the recorded data (Mellor et al., 2023). This is supported by our observation that trials with lower correction performance were associated with elevated signal power and increased participant movement. A potential implication is that CDM estimation and CFA correction performance may benefit from restricting the dataset to periods of limited movements, while allowing participants to move between trials. Alternatively, future studies could explore the integration of movement correction into the processing pipeline.

Finally, there is natural physiological variability between cardiac cycles. For example, in our empirical data, the T-wave, which corresponds to ventricular repolarization, occurred on average 258 ms after the R-peak with a standard deviation of 14.39 ms. Consequently, trial averaging the data to the R-peak will have maximized CDM estimation accuracy for the R-peak, but reduced accuracy for other time points in the cardiac cycle. Future studies may extend the method by constructing separate CDM estimates for each of the key time points in the cardiac cycle (P, Q, R, S, T waves) and combining these estimates to achieve improved CFA correction.

## 6 Conclusion

In the present study, we developed a model-based cardiac field artefact (CFA) correction method for optically pumped magnetoencephalography (OP-MEG). Our method exploits the opportunity that relatively free head movement in neuroimaging studies using OP-MEG allows for the modelling of the heart’s electrical activity in a torso-centred reference frame, which is minimally confounded by concurrent neuronal activity in the brain.

Through simulations and an empirical proof-of-principle demonstration, we showed that the method accurately predicts and reduces sensor-level CFA, while remaining robust to inaccuracies in the forward model. This facilitates application in experimental settings where detailed anatomical models of the participant’s torso may not be available.

The method provides a foundation for OP-MEG studies investigating neural processes that are intrinsically linked to cardiac activity, including cardiac interoception. More broadly, our work demonstrates how greater movement freedom in naturalistic imaging methods can be exploited to create model-based approaches for physiological artefact correction.

## Supplementary Material

Supplementary Material

## Data Availability

The analysis code is publicly available on Zenodo (https://zenodo.org/records/20209782). The corresponding dataset is publicly available on Zenodo (https://zenodo.org/records/20202674).
