# Supplementary material for: Model-based cardiac field artefact correction for OP-MEG
Source: Imaging Neurosci (Camb). 2026 Sep 3;4:IMAG.a.1353. doi: 10.1162/IMAG.a.1353 (PMC13543444; doi:10.1162/IMAG.a.1353)
Supplement: Supplementary file 1 [file IMAG.a.1353_supp.pdf]

Model-Based Cardiac Field Artefact Correction for OP-MEG

Supplementary Materials

Sascha P. Woelk[1†], Nicholas A. Alexander[2], George C. O'Neill[3], Sarah N. Garfinkel[1], Gareth R. Barnes[2]

[1] Institute of Cognitive Neuroscience, University College London, UK
[2] Department of Imaging Neuroscience, Institute of Neurology, University College London, London, UK
[3]Department of Neuroscience, Physiology and Pharmacology, University College London, UK
[†]Corresponding author

Supplementary Material 1

To assess the impact of omitting the head from the volume conductor model, we compared the CDM estimates obtained using a thorax-only model to those obtained using a model that included the head. A test dataset was generated, using the head-including volume conductor model, by simulating a single heartbeat overlaid on Gaussian white noise scaled to 10% of the maximum absolute amplitude of the simulated cardiac signal (i.e. the reference signal). This dataset was then corrected twice: once using a CDM estimate obtained with the thorax-only model and once using a CDM estimate obtained with the head-including model. Both models yielded similar correction accuracy, reducing the simulated cardiac signal to less than 1% of its original amplitude. The mean absolute residual was 0.67% of the reference signal for correction with the thorax-only model, compared to 0.85% of the reference signal for the head-including model, although this difference was not statistically significant ($t(74) = -1.76$,

p = .082).

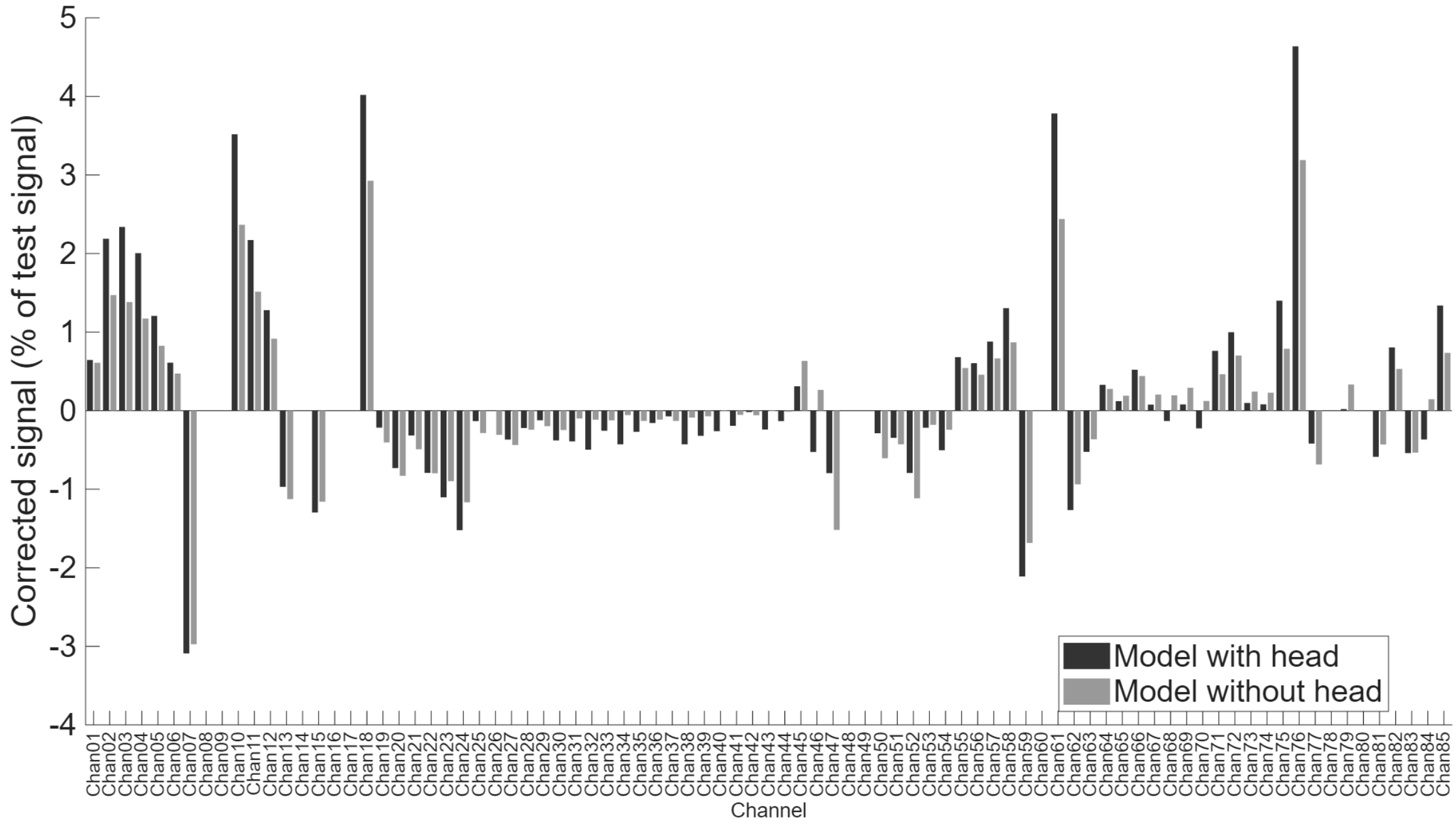

Fig. S1.1. Relative CFA-correction performance of CDM estimates obtained using a thorax-only volume conductor model and a model including the head. *The test signal was a single heartbeat simulated with a torso model including the head. Residual amplitudes for correction with CDM estimates derived from either a torso-including or a thorax-only volume conductor model are shown side by side. Correction errors were computed on a sensor-by-sensor basis relative to each sensor's own reference signal amplitude; sensors with amplitudes below 5% of the maximum absolute reference signal amplitude were excluded to avoid unstable ratios.*

Supplementary Material 2

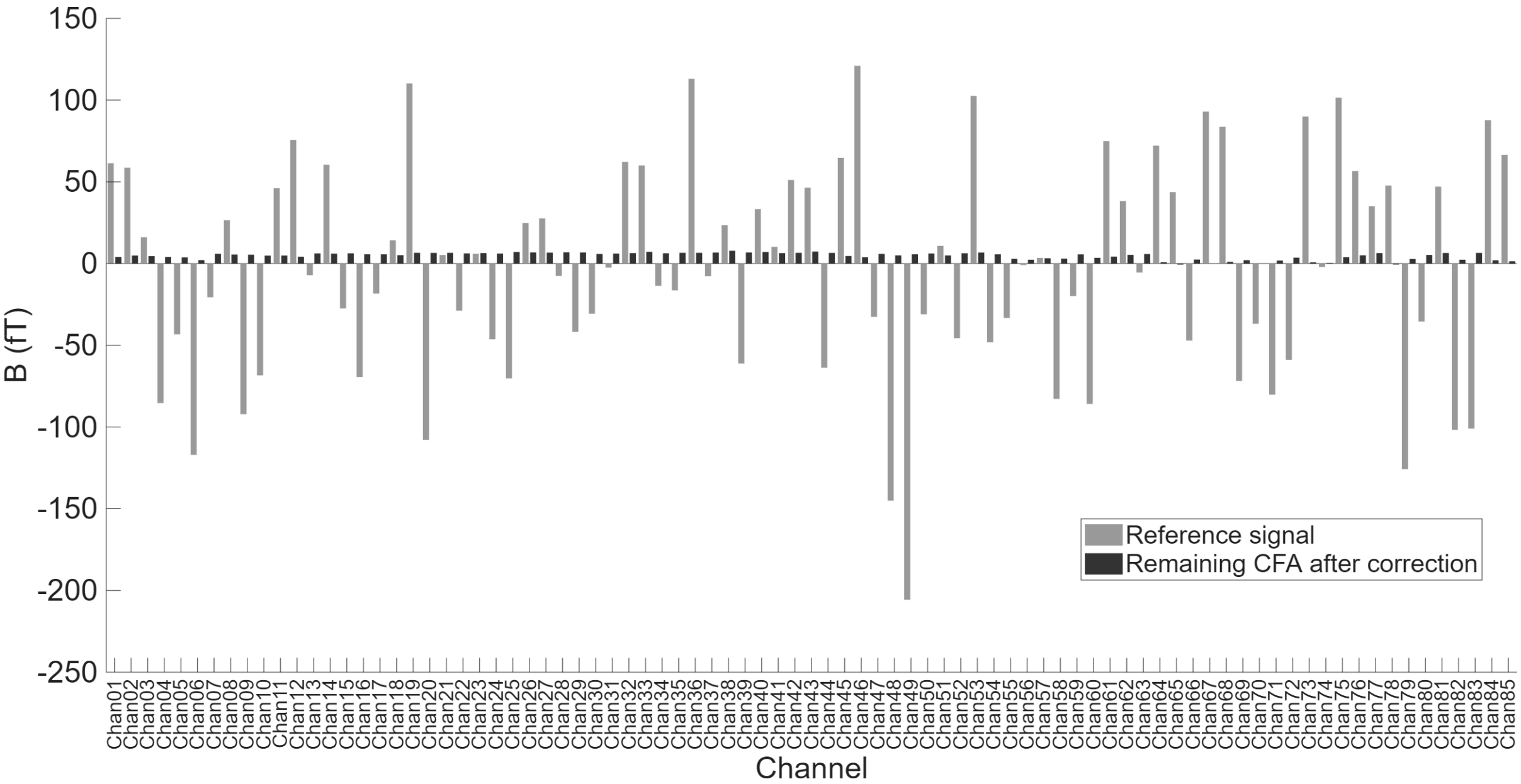

Fig. S2.1. Simulated OP-MEG data before and after CFA-correction, relative to the reference signal. *The reference signal was scaled to 10% of the absolute peak amplitude of the simulated cardiac signal, consistent with previous research showing that cardiac magnetic fields typically exceed cortical signals by approximately one order of magnitude (Hämäläinen et al., 1993). Bars represent measured field amplitudes per OP-MEG channel at time of the R-peak of the cardiac cycle. Light grey bars show the reference signal (scaled to 10% of the peak amplitude of the CFA to match the amplitude of a typical evoked field). Dark grey bars show the remnant CFA after correction with the CDM estimate. The CDM was estimated with data from a single head rotation.*

Supplementary Material 3

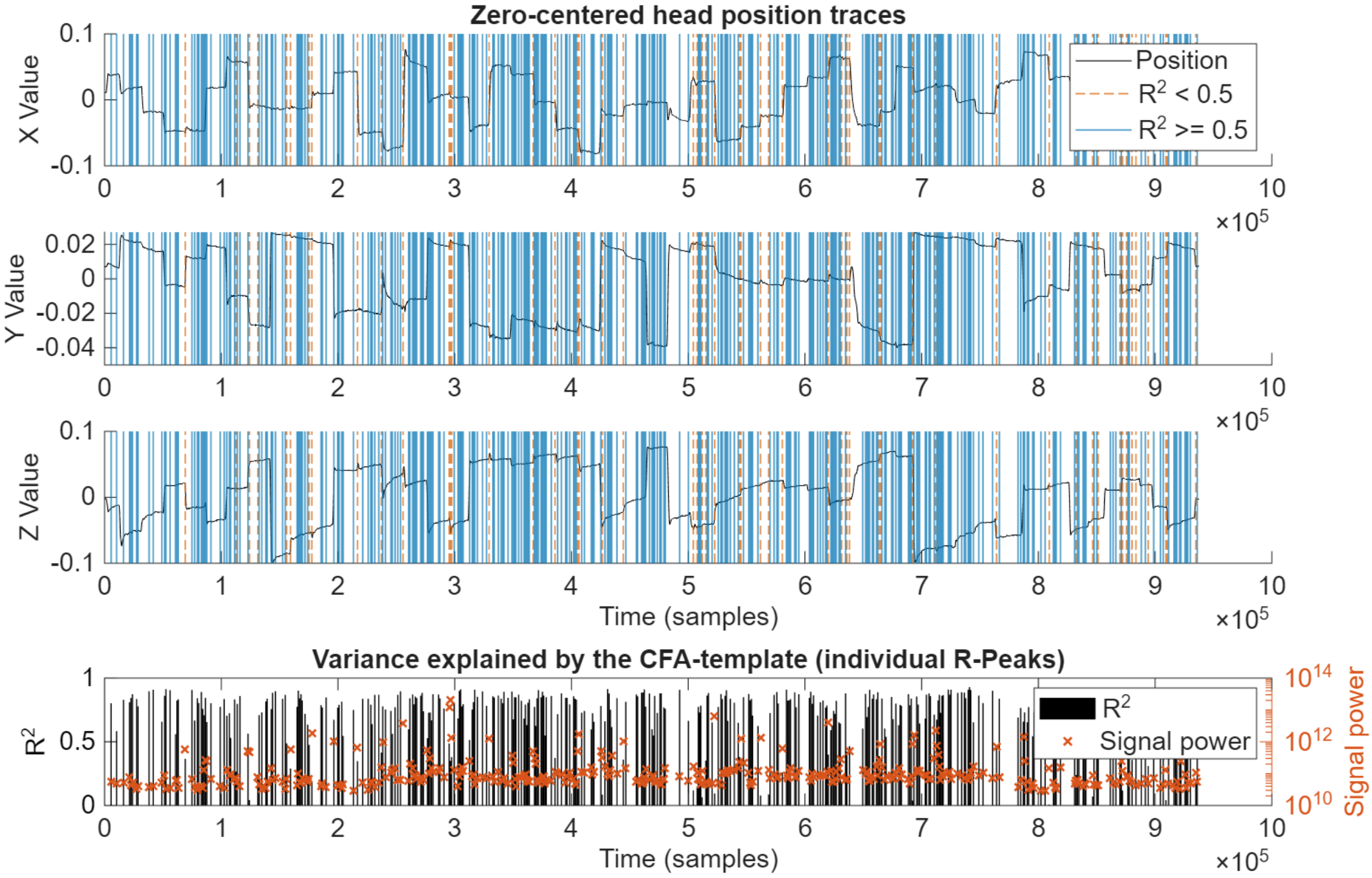

Fig. S3.1. CFA-correction performance on a single-trial level for the silent recording with head rotation. *Performance was measured as the correlation between the predicted CFA and the uncorrected data at time of the R-peak (N = 367 trials). Top three panels: head positions (x, y, z) throughout the recording. R-peaks with high/low variance explained (R^2 <0.5 / R^2>=0.5) are indicated by vertical lines (orange/blue). Bottom panel: CFA-correction performance (variance explained) for all R-peaks in the recording. Crosses indicate the signal power in the window 200 ms before to 200 ms after each R-peak.*

Supplementary Material 4

Distributions and full results tables of bootstrapped evaluation metrics comparing corrected signals (average sensor amplitudes in the N100 time window) and the ground truth (estimated from artefact-free trials).

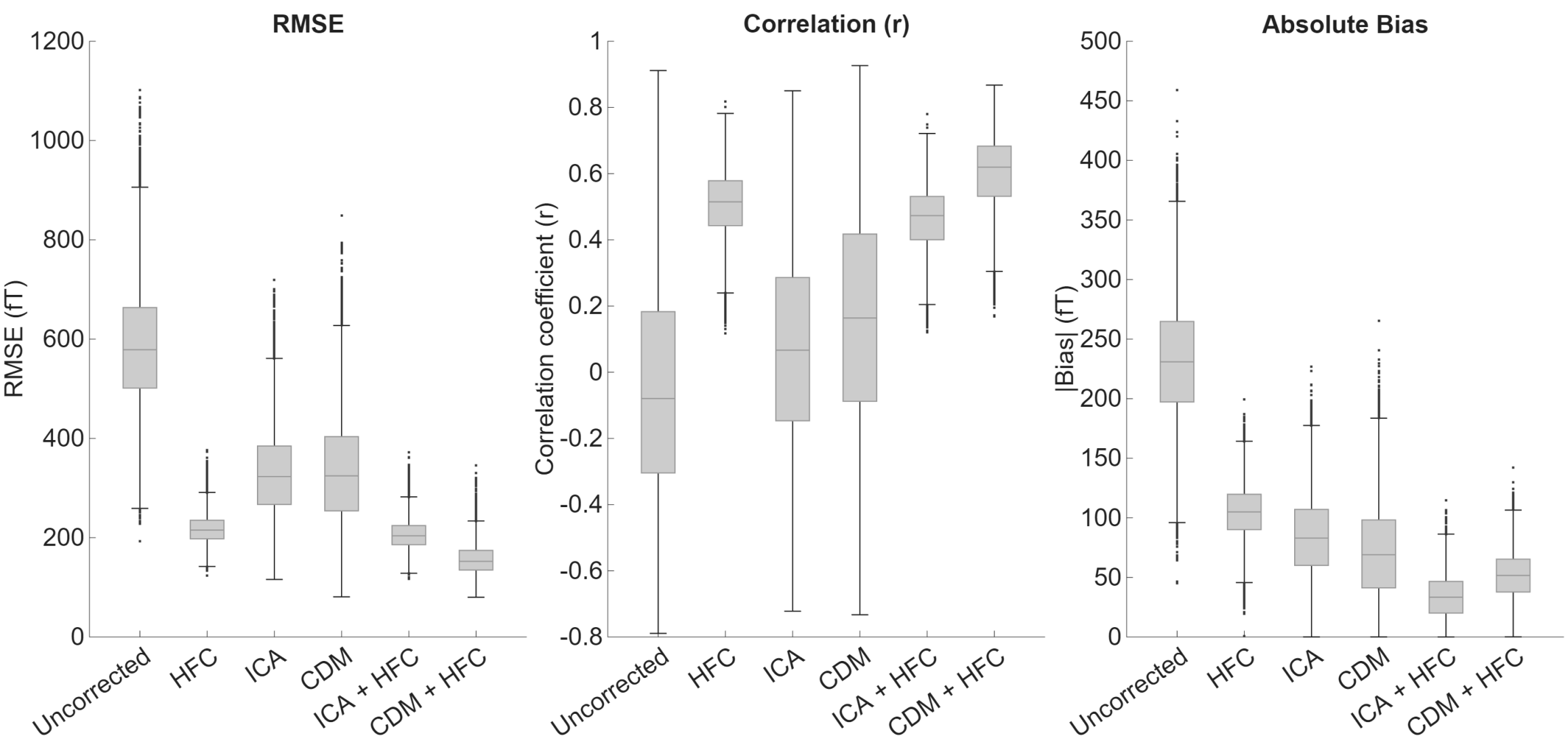

***Fig. S4.1. Bootstrap distributions of sensor-level N100 reconstruction metrics for the facing-forward recording**. Boxes indicate the interquartile range, centre lines the median, whiskers extend to the most extreme values within 1.5 interquartile ranges, and points indicate individual bootstrap samples.*

Table A4.1. Facing-forward: Sensor-level evaluation metrics relative to the ground truth N100 estimate

Method	RMSE (fT)	RMSE 95% CI	Correlation r	r 95% CI	Absolute bias (fT)	Absolute bias 95% CI
**Uncorrected**	585.43	[367.77, 838.78]	-.050	[-.587, .615]	231.47	[134.22, 332.02]
**HFC**	217.53	[166.58, 280.42]	.507	[.298, .675]	105.08	[61.73, 149.18]

**ICA**	330.76	[186.45, 521.62]	.070	[-.465, .608]	83.87	[18.06, 152.76]
**CDM**	334.19	[150.78, 570.57]	.163	[-.453, .760]	71.31	[5.17, 154.30]
**ICA + HFC**	206.41	[155.12, 272.00]	.463	[.259, .622]	34.03	[2.49, 72.06]
**CDM + HFC**	156.83	[108.53, 229.91]	.601	[.353, .768]	51.69	[12.00, 91.80]

Table A4.2. Facing-forward: Paired bootstrap differences (i.e. relative improvement versus the uncorrected test data)

**Comparison**	**Mean difference**	**95% CI**
**HFC - Test, RMSE (fT)**	-367.90	[-578.34, -181.93]
**HFC - Test, Δr**	0.556	[-.104, .952]
**HFC - Test, absolute bias (fT)**	-126.39	[-204.72, -51.72]
**ICA - Test, RMSE (fT)**	-254.67	[-382.68, -122.88]
**ICA - Test, Δr**	0.120	[-.103, .424]
**ICA - Test, absolute bias (fT)**	-147.60	[-205.02, -87.77]
**CDM - Test, RMSE (fT)**	-251.24	[-350.66, -149.68]
**CDM - Test, Δr**	0.213	[-.018, .533]
**CDM - Test, absolute bias (fT)**	-160.16	[-196.12, -117.81]
**ICA + HFC - Test, RMSE (fT)**	-379.02	[-585.20, -198.53]
**ICA + HFC - Test, Δr**	0.513	[-.099, .877]
**ICA + HFC - Test, absolute bias (fT)**	-197.45	[-286.20, -110.94]
**CDM + HFC - Test, RMSE (fT)**	-428.61	[-634.84, -242.89]
**CDM + HFC - Test, Δr**	0.651	[.033, .979]
**CDM + HFC - Test, absolute bias (fT)**	-179.78	[-264.46, -98.82]

Table A4.3. Facing-forward: Key paired bootstrap comparisons involving CDM + HFC

**Comparison**	**Mean difference**	**95% CI**
**CDM + HFC - CDM, RMSE (fT)**	-177.36	[-369.43, -24.22]
**CDM + HFC - CDM, Δr**	0.438	[-.093, .846]
**CDM + HFC - CDM, absolute bias (fT)**	-19.62	[-87.57, 38.63]
**CDM + HFC - HFC, RMSE (fT)**	-60.70	[-91.36, -33.32]
**CDM + HFC - HFC, Δr**	0.095	[-.006, .210]

**CDM + HFC - HFC, absolute bias (fT)**	-53.39	[-64.42, -42.45]
**CDM + HFC - ICA, RMSE (fT)**	-173.93	[-323.15, -55.65]
**CDM + HFC - ICA, Δr**	0.531	[.037, .876]
**CDM + HFC - ICA, absolute bias (fT)**	-32.18	[-85.02, 17.64]

Table A4.4. Facing-forward: Key paired bootstrap comparisons involving ICA + HFC

**Comparison**	**Mean difference**	**95% CI**
**ICA + HFC - CDM, RMSE (fT)**	-127.78	[-327.51, 31.53]
**ICA + HFC - CDM, Δr**	0.300	[-.241, .756]
**ICA + HFC - CDM, absolute bias (fT)**	-37.28	[-107.49, 22.37]
**ICA + HFC - HFC, RMSE (fT)**	-11.12	[-23.15, -0.14]
**ICA + HFC - HFC, Δr**	-0.044	[-.135, .065]
**ICA + HFC - HFC, absolute bias (fT)**	-71.06	[-90.56, -50.72]
**ICA + HFC - ICA, RMSE (fT)**	-124.35	[-279.28, -0.41]
**ICA + HFC - ICA, Δr**	0.393	[-.101, .775]
**ICA + HFC - ICA, absolute bias (fT)**	-49.84	[-103.53, 1.63]
**ICA + HFC - CDM + HFC, RMSE (fT)**	49.58	[26.66, 75.87]
**ICA + HFC - CDM + HFC, Δr**	-0.138	[-.210, -.072]
**ICA + HFC - CDM + HFC, absolute bias (fT)**	-17.66	[-27.12, -5.77]

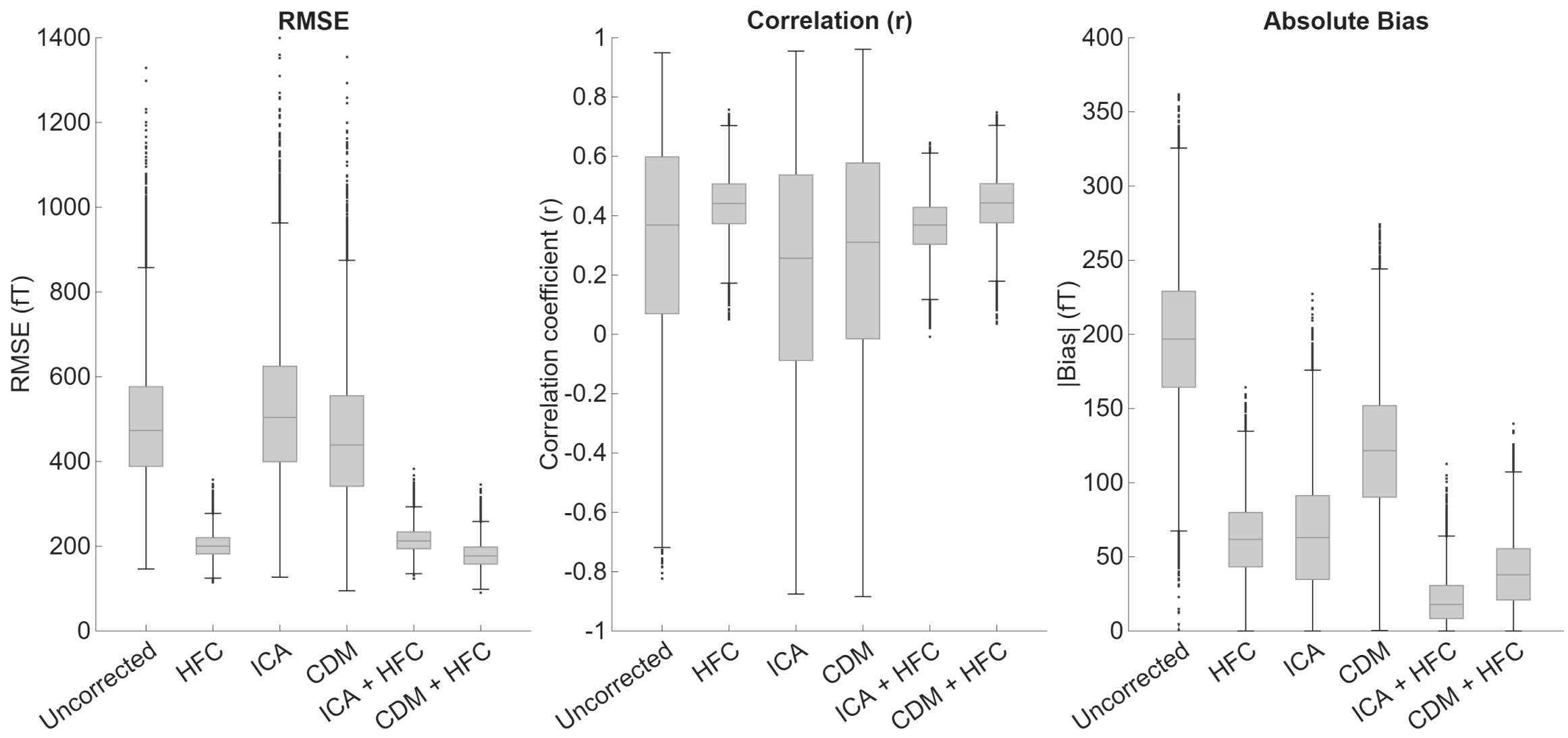

***Fig. S4.2. Bootstrap distributions of sensor-level N100 reconstruction metrics for the recording with intermittent head rotation**. Boxes indicate the interquartile range, centre lines the median, whiskers extend to the most extreme values within 1.5 interquartile ranges, and points indicate individual bootstrap samples.*

Table A4.5. Head-rotation: Sensor-level evaluation metrics relative to the ground truth N100

**Method**	**RMSE (fT)**	**RMSE 95% CI**	**Correlation r**	**r 95% CI**	**Absolute bias (fT)**	**Absolute bias 95% CI**
**Uncorrected**	491.53	[264.11, 816.12]	.313	[-.456, .835]	196.37	[99.85, 291.53]
**HFC**	202.31	[152.37, 264.78]	.438	[.239, .617]	61.94	[11.13, 114.62]
**ICA**	520.58	[245.93, 892.63]	.212	[-.578, .822]	65.36	[4.11, 147.37]
**CDM**	458.15	[206.34, 813.19]	.265	[-.522, .848]	121.29	[30.50, 211.68]
**ICA + HFC**	215.25	[163.92, 281.16]	.363	[.168, .525]	21.19	[0.86, 59.09]
**CDM + HFC**	179.71	[129.06, 246.51]	.439	[.232, .617]	39.48	[2.30, 89.42]

Table A4.6. Head-rotation: Paired bootstrap differences (i.e. relative improvement versus the uncorrected test data)

Comparison	Mean difference	95% CI
**HFC - Test, RMSE (fT)**	-289.22	[-572.04, -79.07]
**HFC - Test, Δr**	0.125	[-.449, .786]
**HFC - Test, absolute bias (fT)**	-134.43	[-207.23, -56.13]
**ICA - Test, RMSE (fT)**	29.05	[-123.06, 157.77]
**ICA - Test, Δr**	-0.101	[-.456, .231]
**ICA - Test, absolute bias (fT)**	-131.01	[-190.46, -61.10]
**CDM - Test, RMSE (fT)**	-33.38	[-123.13, 44.36]
**CDM - Test, Δr**	-0.048	[-.315, .243]
**CDM - Test, absolute bias (fT)**	-75.07	[-101.81, -47.94]
**ICA + HFC - Test, RMSE (fT)**	-276.28	[-553.71, -70.73]
**ICA + HFC - Test, Δr**	0.050	[-.470, .679]
**ICA + HFC - Test, absolute bias (fT)**	-175.18	[-271.78, -59.20]
**CDM + HFC - Test, RMSE (fT)**	-311.82	[-590.60, -100.80]
**CDM + HFC - Test, Δr**	0.127	[-.430, .763]
**CDM + HFC - Test, absolute bias (fT)**	-156.89	[-232.78, -73.80]

Table A4.7. Head-rotation: Key paired bootstrap comparisons involving CDM + HFC

Comparison	Mean difference	95% CI
**CDM + HFC - CDM, RMSE (fT)**	-278.44	[-588.39, -45.04]
**CDM + HFC - CDM, Δr**	0.175	[-.435, .810]
**CDM + HFC - CDM, absolute bias (fT)**	-81.81	[-154.51, -4.74]
**CDM + HFC - HFC, RMSE (fT)**	-22.60	[-42.61, -5.99]
**CDM + HFC - HFC, Δr**	0.001	[-.047, .060]
**CDM + HFC - HFC, absolute bias (fT)**	-22.46	[-31.94, 3.77]
**CDM + HFC - ICA, RMSE (fT)**	-340.87	[-667.28, -81.18]
**CDM + HFC - ICA, Δr**	0.228	[-.422, .878]
**CDM + HFC - ICA, absolute bias (fT)**	-25.87	[-93.41, 32.54]

Table A4.8. Head-rotation: Key paired bootstrap comparisons involving ICA + HFC

Comparison	Mean difference	95% CI

**ICA + HFC - CDM, RMSE (fT)**	-242.91	[-547.10, -14.94]
**ICA + HFC - CDM, Δr**	0.098	[-.476, .724]
**ICA + HFC - CDM, absolute bias (fT)**	-100.11	[-191.34, 12.26]
**ICA + HFC - HFC, RMSE (fT)**	12.93	[-5.06, 29.13]
**ICA + HFC - HFC, Δr**	-0.075	[-.191, .049]
**ICA + HFC - HFC, absolute bias (fT)**	-40.76	[-82.36, 46.39]
**ICA + HFC - ICA, RMSE (fT)**	-305.33	[-625.92, -53.90]
**ICA + HFC - ICA, Δr**	0.152	[-.461, .792]
**ICA + HFC - ICA, absolute bias (fT)**	-44.17	[-128.20, 39.69]
**ICA + HFC - CDM + HFC, RMSE (fT)**	35.54	[13.42, 58.07]
**ICA + HFC - CDM + HFC, Δr**	-0.076	[-.170, .013]
**ICA + HFC - CDM + HFC, absolute bias (fT)**	-18.30	[-54.58, 45.64]

Supplementary Material 5

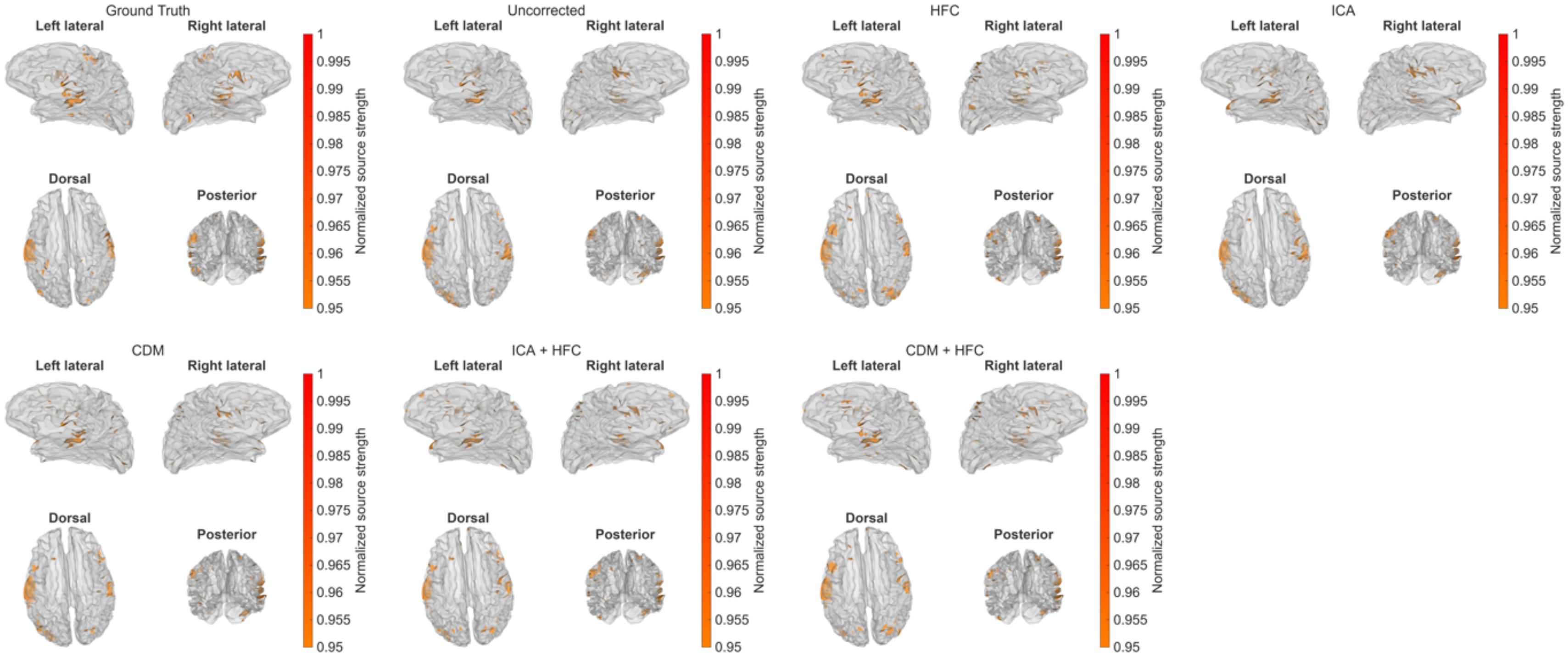

Fig. S5.1. Estimated distributed source solution for the auditory N100 response during the facing-forward recording. *From top-left to bottom-right: ground truth (i.e. N100 estimated from artefact-free trials); uncorrected data with cardiac artefact; homogeneous field correction (HFC); independent component analysis (ICA); cardiac dipole moment estimate (CDM); ICA correction followed by HFC; CDM followed by HFC. Source estimates are shown as cortical surface maps within the expected N100 window (i.e. 80-120 ms post-stimulus). Values represent absolute source amplitude, normalised across the cortex within each dataset, with only the strongest 5% of source locations displayed.*

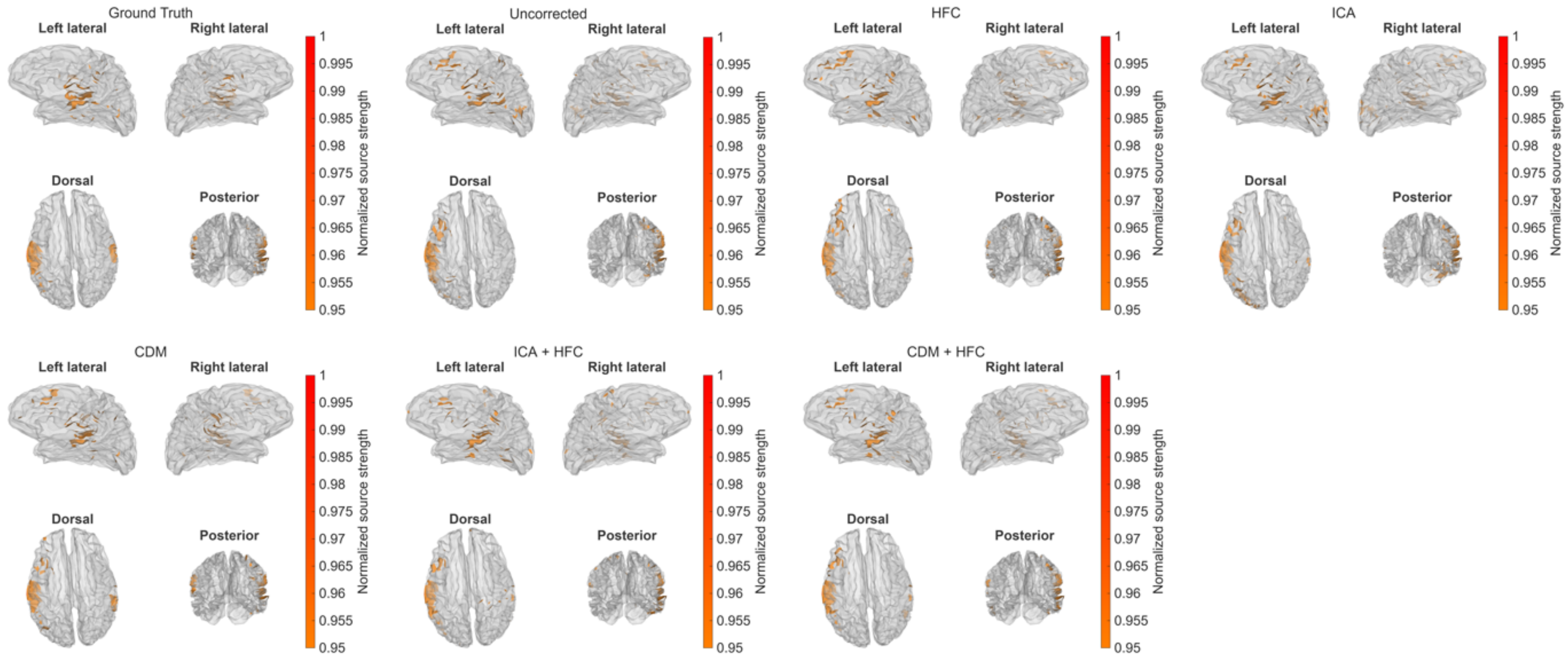

Fig. S5.2. Estimated distributed source solution for the auditory N100 response during the head-rotation recording. *From top-left to bottom-right: ground truth (i.e. N100 estimated from artefact-free trials); uncorrected data with cardiac artefact; homogeneous field correction (HFC); independent component analysis (ICA); cardiac dipole moment estimate (CDM); ICA correction followed by HFC; CDM followed by HFC. Source estimates are shown as cortical surface maps within the expected N100 window (i.e. 80-120 ms post-stimulus). Values represent absolute source amplitude, normalised across the cortex within each dataset, with only the strongest 5% of source locations displayed.*
